# The evolution of eavesdropping on heterospecific alarm calls: Relevance, reliability, and personal information

**DOI:** 10.1002/ece3.10272

**Published:** 2023-07-12

**Authors:** Cameron Rouse Turner, Matt Spike, Robert D. Magrath

**Affiliations:** ^1^ Computational Cognitive Science Lab, Department of Computer Science Princeton University Princeton New Jersey USA; ^2^ Division of Ecology and Evolution, Research School of Biology Australian National University Canberra Australian Capital Territory Australia; ^3^ Centre for Language Evolution, School of Philosophy, Psychology and Language Sciences The University of Edinburgh Edinburgh UK

**Keywords:** alarm call, eavesdropping, signal detection theory, signaling, social information use

## Abstract

Interceptive eavesdropping on the alarm calls of heterospecifics provides crucial information about predators. Previous research suggests predator discrimination, call relevance, reliability, and reception explain when eavesdropping will evolve. However, there has been no quantitative analysis to scrutinize these principles, or how they interact. We develop a mathematical framework that formalizes the study of the key principles thought to select for eavesdropping. Interceptive eavesdropping appears to be greatly affected by the threat faced by caller and eavesdropper, as well as presence of informational noise affecting the detection of calls and predators. Accordingly, our model uses signal detection theory to examine when selection will favor alarm calling by a sender species and fleeing by an eavesdropping receiver species. We find eavesdropping is most strongly selected when (1) the receiver faces substantial threats, (2) species are ecologically similar, (3) senders often correctly discriminate threats, (4) receivers often correctly perceive calls, and (5) the receiver's personal discrimination of threats is poor. Furthermore, we find (6) that very high predation levels can select against eavesdropping because prey cannot continuously flee and must conserve energy. Reliability of heterospecific calls for identifying threats is thought to be important in selecting for eavesdropping. Consequently, we formally define reliability, showing its connection to specificity and sensitivity, clarifying how these quantities can be measured. We find that high call relevance, due to similar vulnerability to predators between species, strongly favors eavesdropping. This is because senders trade‐off false alarms and missed predator detections in a way that is also favorable for the eavesdropper, by producing less of the costlier error. Unexpectedly, highly relevant calls increase the total number of combined errors and so have lower reliability. Expectedly, when noise greatly affects personally gathered cues to threats, but not heterospecific calls or detection of predators, eavesdropping is favored.

## Graphical abstract

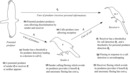

## 1 INTRODUCTION

### 1.1 Eavesdropping on heterospecific alarm calls

Information is critical for animals to make adaptive decisions, including about how to avoid predators. Information can come from personal assessment of the environment or the inadvertent cues and communicatory signals of others (Danchin et al., 2004; Dall et al., 2005). Animals produce *inadvertent cues* from their routine activities, which can be a source of information, such as when a frog hears the flapping of an approaching predatory bat (Bernal et al., 2007); (Table 1 provides a glossary of key terms). By contrast, *communicatory signals* are produced deliberately to enable communication with intended receivers (Bradbury & Vehrencamp, 2011; Maynard Smith & Harper, 2003; Searcy & Nowicki, 2005). However, signals intended for specific receivers can also be detected by third parties in the process of *eavesdropping*.

**TABLE 1:** Glossary of key terms.

Signal (communicatory)	Traits that are selected because the sender benefits from the intended receiver's response, and the receiver benefits from responding to the signal. That is, signal evolution is driven by sender–receiver interaction. In our model: Alarm calls
Signal (Signal detection theory)	Information or evidence that an event has occurred (an index of the state of the environment)
Inadvertent cue	Information produced by an individual that can affect the behavior of another but has not evolved for that purpose. (The term inadvertent cue is sometimes used even when there has been selection for concealment)
Cue (Signal detection theory)	Any observable data; inclusive of both informational signal and noise
Eavesdropping	The use of communicatory signals by a third party who is not the intended recipient, for whom these signals are inadvertent cues. We use the term to mean **interceptive eavesdropping** as opposed to **social eavesdropping**. Inadvertent cue use is only eavesdropping when it is on a communicatory signal
Reliability	The degree to which a sender calls only (no false alarms) and always (no missed detections) when a predator is present, and not when they are absent. **Eavesdropping reliability** refers to how well calls correctly categorize predators of the receiver. **Sender reliability** refers to how well calls correctly categorize predators of the sender
Discrimination	The degree to which a sender species correctly detects a predator being present
Reception	The degree to which the sender's calls are correctly detected by the receiver
Relative vulnerability	The degree to which an individual's fitness is reduced by predation, accounting also for costs of fleeing. The relative vulnerability is higher when the predator is more dangerous, there is little cost of fleeing and little benefit to remaining in place. For the receiver, the relative vulnerability also accounts for the probability the predator is shared or unique; so that the relative vulnerability of the receiver is high when they face substantial risks from a shared predator
Relevance	The degree to which a sender's call refers to a predator to which the receiver is similarly vulnerable. **High relevance** implies closeness of relative vulnerability, selecting for eavesdropping. **Low relevance** implies the sender produces too many false alarms for the receiver, because the sender is more vulnerable. **Inadequate relevance** implies the sender has too many missed detections for the receiver, so inadequately identifies the receiver's predators, because the sender is less vulnerable
Correct categorisations	Either a **correct detection** of an event leading to an appropriate response, or a **correct rejection**, leading to appropriately not responding because an event has not occurred. A correct categorization depends on the focal individual, such that a correct categorization of a predator of the sender may not correspond to a correct categorization of a receiver's predator
Errors	Either a **false alarm**, resulting in an inappropriate response because of the incorrect assessment that an event has occurred, or a **missed detection** resulting in a lack of a response because the event was assessed as not occurring when it in fact did occur. As with correct categorizations, errors are relative to the event considered
Noise (Signal detection theory)	Informational interference that either distorts signals leading to missed detections, or resembles signals, leading to false alarms
Detection threshold	A threshold set by an individual that determines the probability of correct categorizations, compared to errors. Individuals respond only to signals whose strength is above the detection threshold. The sender has a detection threshold for predators, while the receiver has a detection threshold for alarm calls
Adaptive biasing	Setting a **detection threshold** to either be **gullible**, which reduces missed detections at the cost of more false alarms, or **fastidious**, which reduces false alarms at the cost of more missed detections

Instances of eavesdropping are classified into two categories: social eavesdropping and interceptive eavesdropping (Peake, 2005). Social eavesdropping occurs when a third party collects information about others from the signals they produce during communication. For instance, Siamese fighting fish (*Betta splendens*) adjust their aggression depending on the signaling and success others have had in observed fights (Oliveira et al., 1998). By contrast, interceptive eavesdropping occurs when a third party uses a signal to infer the state of the environment. Social eavesdropping may often affect the signalers' fitness and is thought to be common among conspecifics; however, the signaler may be unaffected by interceptive eavesdropping, which commonly involves heterospecifics (Peake, 2005). Interceptive eavesdropping often entails communicatory signals that are broadcast to intended receivers over a large area, so may also reach eavesdroppers for whom these communicatory signals are inadvertent cues.

We focus on interceptive eavesdropping on the *alarm calls* of other species, which occurs across diverse taxa, and is an ecologically critical source of information about danger (Magrath et al., 2015). Alarm calls are usually broadcast to signal danger to conspecifics, but are also relevant to eavesdropping heterospecifics that are vulnerable to similar predators (Caro, 2005; Goodale et al., 2010; Magrath et al., 2015, 2020; Schmidt et al., 2010; Seppänen et al., 2007). Our focus is on alarm calls that prompt fleeing, and not other responses like mobbing. We derive conditions for heterospecific eavesdropping, considering both the selection for alarms by callers (*senders*) and their reception by listeners (*receivers*). Although information about predator danger is crucial, some species disregard heterospecific alarm calls that would seemingly be beneficial, and this requires explanation. For instance, scimitarbills (*Rhinopomastus cyanomelas*) and pied babblers (*Turdoides bicolor*) have common predators, yet while scimitarbills eavesdrop on babblers, the reverse is not observed (Ridley et al., 2014).

### 1.2 Reliability and relevance may constrain eavesdropping

The reliability of heterospecific alarm calls is thought to be an important constraint in the evolution of eavesdropping (Goodale et al., 2010; Magrath et al., 2015; Schmidt et al., 2010; Seppänen et al., 2007). A calling species is reliable to the degree it only and always calls when a predator is present (Magrath et al., 2015; Searcy & Nowicki, 2005). In other words, reliability is high if there are few false alarms given to non‐predators (false positives), as well as few missed detections of predators (false negatives). Therefore, reliable alarms have both good sensitivity and specificity (sensu Goodale et al., 2010).

Later, we will show that there are two broad types of reliability, depending on if calls correspond to a predator of the caller or the potential eavesdropper. *Sender reliability* is the degree to which senders make few errors in calling with regards to their own predators. *Eavesdropping reliability* is defined from the receiver's perspective and is the degree to which senders make few errors in calling in response to the receiver's predators. Sender reliability will differ from eavesdropping reliability depending on how often predators that are encountered are shared between the species or unique to one. Reliability is argued to be critical for both senders and receivers because missing predators is dangerous, and unnecessary fleeing is costly in expending energy and forgoing other opportunities.

Eavesdroppers should benefit most from heterospecific alarm calls when they are relevant (Magrath et al., 2015; Searcy & Nowicki, 2005). Call *relevance* is the degree to which heterospecific alarm calls identify threats that pose similar levels of danger to the eavesdropper as they do to the sender. Relevance is subtly different from eavesdropping reliability, because relevance refers to the precise similarity of fitness consequences of encounters with predators for sender and receiver, whereas eavesdropping reliability refers only to the correspondence between calls and the presence of the receiver's predators. Later, we will formally clarify the relationship between these related principles.

As expected, individuals are more likely to respond to more relevant heterospecific alarm calls (Magrath et al., 2009; Meise et al., 2018; Munoz et al., 2015; Palmer & Gross, 2018; Rainey et al., 2004a, 2004b). This includes different alarm calls from the same calling species. For example, hornbills (*Ceratogymna* spp) respond to alarm calls of monkeys (*Cercopithecus* spp) that warn of crowned eagles (*Stephanoaetus coronatus*), which also prey on hornbills, but not to monkey alarm calls warning of leopards (*Panthera pardus*), which rarely threaten these birds (Rainey et al., 2004a, 2004b). Furthermore, less vulnerable species appear less likely to eavesdrop on more vulnerable species, because the more vulnerable species produce a greater number of false alarms that would cause unnecessary fleeing from the perspective of the less vulnerable species (Magrath et al., 2009; Munoz et al., 2015; Palmer & Gross, 2018; Rainey et al., 2004a, 2004b). Broadly, relevance may be important because an eavesdropping species can more effectively use information from another species to stand‐in for information from their own species (Goodale et al., 2010; Meise et al., 2018; Seppänen et al., 2007).

### 1.3 Noise affects transmission and may constrain eavesdropping

Even for species that are vulnerable to the same suite of predators, a calling species may produce errors due to limitations in *discrimination* of predators, reducing the net benefit of eavesdropping. Discrimination of predators is hindered by informational interference from the environment that can distort or resemble cues from a predator and lead to incorrect categorization of cues as innocuous or dangerous. This interference is referred to as *noise* in signal detection theory (Wiley, 2006, 2013, 2015, 2017). A common source of noise leading to errors in caller discrimination are harmless animals that resemble predators (Gyger et al., 1987). Species also vary in their ability to spot danger and call to real threats, due to sensory systems or location. For example, animals that forage on the ground, or near obscuring cover, are less able to spot predators than those in higher positions with a clearer view (Martínez et al., 2018; Martínez & Zenil, 2012; McLachlan et al., 2019; Radford et al., 2009). By decreasing the number of errors, good discrimination should result in more calls corresponding to predators and select for eavesdropping.

Even if calls often indicate a threat, eavesdroppers can only use that information if they can perceive heterospecific alarm calls. Therefore, call *reception*, defined as how well calls are detected by heterospecifics, should also affect the evolution of eavesdropping (Magrath et al., 2015). Similar to predator discrimination, informational noise also affects how well alarm calls are received by a potential eavesdropper. This is particularly important for eavesdropping, as individuals may receive distant calls, not originating from an immediate social group, increasing the potential for errors due to signal attenuation and degradation during transmission (Murray & Magrath, 2015; Seppänen et al., 2007). Furthermore, sensory systems can be tuned to conspecific signals, meaning that heterospecific signals may be more difficult to perceive (Murray & Magrath, 2015). Compounding the problem of perception, background noise can make calls difficult to hear or identify, particularly at a distance. True alarm calls may be masked by noise, or non‐alarm calls mistaken for useful alarms (Grade & Sieving, 2016; Morris‐Drake et al., 2017; Templeton et al., 2016; Zhou et al., 2019). Too many reception errors could counteract the benefits of eavesdropping.

### 1.4 Personal information may constrain eavesdropping

In addition to gaining social information from eavesdropping, receivers may use personal information gained from the sights, smells, and sounds of predators. Broadly, individuals should use superior sources of information when there are alternatives available (Dall et al., 2005; Danchin et al., 2004). Therefore, eavesdropping may not be selected if personal information allows good inferences, whereas poor personal information favors eavesdropping (Jones & Sieving, 2019; Martínez & Zenil, 2012; McLachlan et al., 2019; Ratnayake et al., 2021). For example, species that glean food from nearby surfaces may be less able to detect threats themselves, and so are more reliant on eavesdropping, than those species that scan for distant prey (Martínez & Zenil, 2012).

### 1.5 Reserve depletion may constrain eavesdropping

Although more frequent encounters with predators should usually favor eavesdropping to avoid threats, when predators are extremely common eavesdropping may be selected against, with prey often choosing to maintain energy reserves rather than flee. Animals face a fundamental trade‐off between energy consumption and avoiding predation when deciding whether to flee (Ferrari et al., 2009; Lima & Bednekoff, 1999; Lima & Dill, 1990; Werner & Anholt, 1993). If predators are common, avoiding predation can be achieved by being cautious and fleeing more often. However, each time prey flee they expend energy and forgo the opportunity to replenish reserves. Therefore, when predators are very frequently encountered, prey may need to be bold, fleeing less often, in order to maintain their energy reserves (Holen & Sherratt, 2021; McNamara & Trimmer, 2019; Trimmer, Ehlman, McNamara, et al., 2017; Trimmer, Ehlman, & Sih, 2017). As a result, eavesdroppers may ignore more alarms when predators are very common and continue foraging.

### 1.6 Modeling approach and aims

We use *signal detection theory* to assess the conditions under which heterospecific eavesdropping can evolve. The conditions necessary for conspecific communication have been given extensive formal treatment and are well understood (Maynard Smith & Harper, 2003). Social eavesdropping has also had some formal treatment (Johnstone, 2001). However, little modeling has directly investigated issues specific to interceptive eavesdropping on heterospecific signals, despite several reviews (Goodale et al., 2010; Magrath et al., 2015, 2020; Schmidt et al., 2010; Seppänen et al., 2007). Signal detection theory provides a well‐developed framework for the quantification of errors and correct categorizations that occur during decision‐making under uncertainty (Green & Swets, 1966; Wickens, 2002) and has been fruitfully deployed in explanations of animal behavior (Dall et al., 2005; Hauber et al., 2020; Holen & Sherratt, 2021; Leavell & Bernal, 2019; McNamara & Trimmer, 2019; Sumner & Sumner, 2020; Trimmer, Ehlman, McNamara, et al., 2017; Trimmer, Ehlman, & Sih, 2017; Wiley, 2006, 2013, 2015, 2017). In signal detection theory, *signal* means any evidence associated with an event or state of the world and so is a broader concept than a signal in animal communication (Table 1). Previous language‐based models about the evolution of eavesdropping invoke concepts that are formalized in signal detection theory (Goodale et al., 2010; Magrath et al., 2015; Schmidt et al., 2010; Seppänen et al., 2007), such as *false alarm*, and empirical tests of alarm call design have drawn predictions from signal detection theory (Tegtman & Magrath, 2020).

**FIGURE 1:**
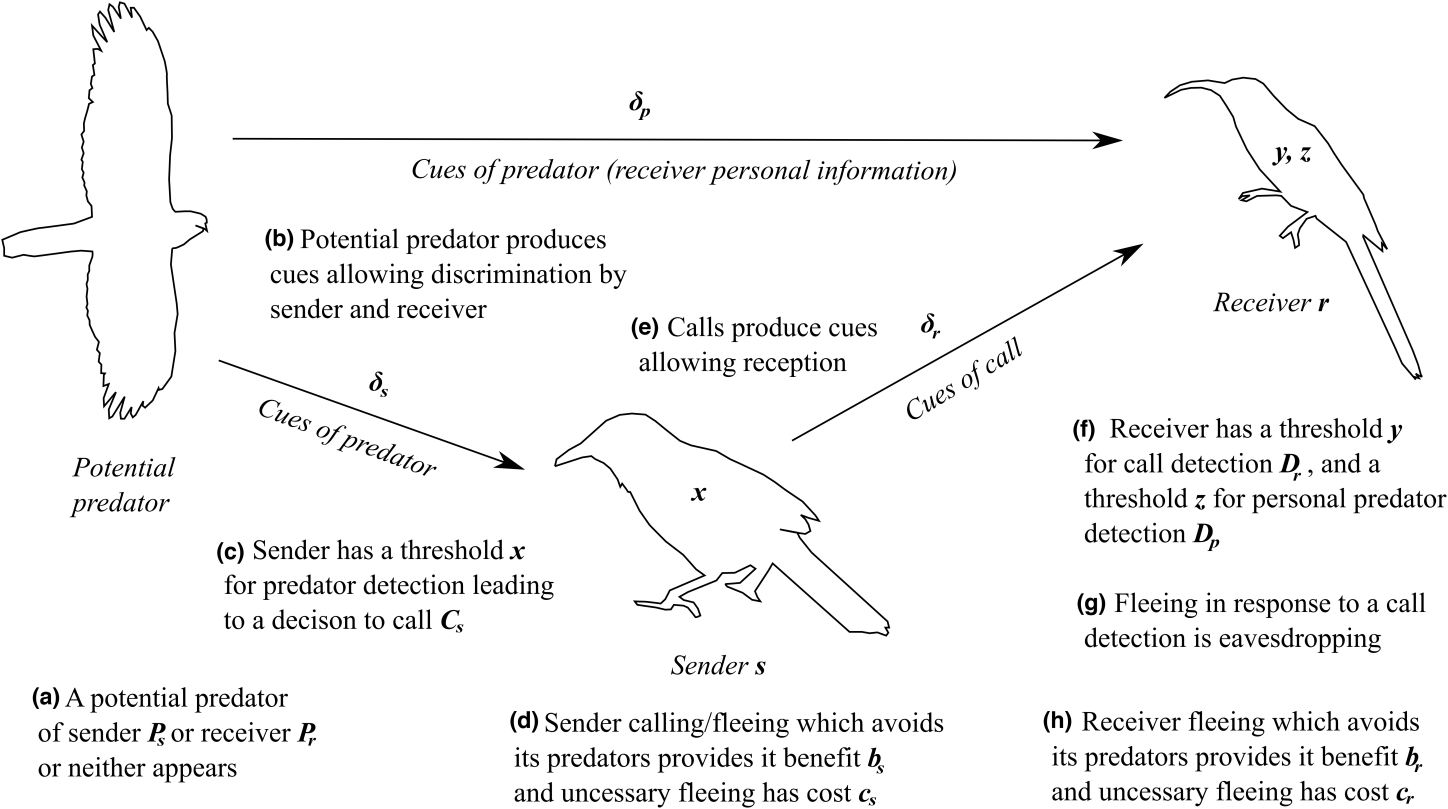
Depiction of elements of the basic model of heterospecific eavesdropping. The flow of information between sender and receiver, and set of possible events in heterospecific eavesdropping, leading to the decision of the receiver to flee.

Here, we develop a mathematical model, based on signal detection theory, to investigate the conditions selecting for eavesdropping on heterospecific alarm calls. The goal of this paper is to scrutinize previous explanations for eavesdropping, as well as compare the influence of different factors on eavesdropping. We model a *sender* species, which gives alarm calls, and a *receiver* species, which can eavesdrop on these calls. This means the sender must resolve the signal detection problem of identifying a predator, whereas the receiver must resolve the signal detection problem of identifying a call. Our model represents a situation in which heterospecific communicatory signals are being used as inadvertent cues by eavesdroppers (Magrath et al., 2009; Meise et al., 2018; Ridley et al., 2014). We assume that senders are not affected by receiver eavesdropping, so deliberate communication with heterospecifics does not evolve. The scope of the model is to explore how eavesdropping is affected by the threats faced by each species and the challenges of using a heterospecific signal as a cue (Goodale et al., 2010; Magrath et al., 2015, 2020; Peake, 2005; Schmidt et al., 2010; Seppänen et al., 2007). The logic of the model is summarized in Figure 1.

We use our model as a formal framework to explore previous theory and observations, which suggest that eavesdropping is selected when there are: (1) high levels of eavesdropping reliability, and (2) predators that pose similar threats to sender and receiver, leading to high call relevance. Furthermore, eavesdropping is likely to be selected when there is less informational noise, such that (3) discrimination and (4) reception are good. Furthermore, (5) eavesdropping should be favored when personal information is poor. Finally, (6) while an increased probability of encountering a predator should generally favor eavesdropping, very high likelihood of encountering a predator should select against eavesdropping. We first describe the model, providing the conditions for the evolution of eavesdropping, consider reliability and its measurement, and then compare our results with expectations from the literature.

## 2 MATERIALS AND METHODS

We use standard evolutionary invasion analysis methods (Otto & Day, 2007), and further explain all derivations in the Appendix S1. Table 2 gives an index of notation.

**TABLE 2:** Index of notation.

Symbol	Description
*x*	The detection threshold for the strength of evidence of a predator above which a sender calls and flees. The sender's threshold that maximizes sender fitness is $\hat{x}$, eavesdropping reliability is ${\hat{x}}_{\rho }$, and selection for eavesdropping is ${\hat{x}}_{r}$
*y, z*	The detection threshold for the strength of evidence of a call *y*, or personal information *z*, above which a receiver will flee
*b*	The benefit of fleeing and avoiding predation
*c*	The cost of unnecessarily fleeing (including missing opportunities of remaining)
*ν*	The relative vulnerability or the risk of predation by a predator of the sender, scaled by the fleeing cost and benefit of safely remaining. In the case of the receiver, this includes the probability that the threat is one of the receiver's predators
P	The probability of a predator being present. *P*_*s*_ denotes a predator of the sender and *P*_*r*_ denotes a predator of the receiver (not mutually exclusive)
*C*_*s*_	A call of the sender is present
*D*_*r*_	Detection by the receiver of a call
*D*_*p*_	Detection by the receiver of a predator using personal information.
*δ*	The distance between the means of distributions of the observed strength of evidence given a signal and noise, and the distribution of strength of evidence of pure noise (sensitivity index). *δ*_*s*_ is the sender's discrimination of predators. *δ*_*r*_ is the receiver's reception of sender calls
*ρ*	Eavesdropping reliability or the total probability of correct categorizations by the sender of threats to the receiver

*Note*: Parameters are subscripted with *s* when they refer to sender and *r* if receiver. P(·) is the probability of an event, and ¬ indicates the event has not occurred. $x,y,\upsilon \in \left(\infty ,\infty \right)$, $b,c\in \left[0,1\right]$, $\delta \in \left[0,\infty \right)$.

### 2.1 Selection for calling decisions by senders

Before being able to extend to the case of an eavesdropping receiver species, we first use signal detection theory to specify the conditions under which senders will produce alarm calls. While there are good reviews of signal detection theory applied ecology (Holen & Sherratt, 2021; Leavell & Bernal, 2019; Sumner & Sumner, 2020), we explain the theory as relevant to our eavesdropping model. The fitness of senders depends on whether a predator of the sender is present or absent (*P*_*s*_ or ¬*P*_*s*_), whether they flee or remain (*C*_*s*_ or ¬*C*_*s*_), and the fitness payoffs associated with the decision to flee. We assume the senders flee and call simultaneously, so they are modeled as the same decision. If an individual flees, they incur a cost due to the energy used. If an individual chooses to remain in place when a predator is present, they risk injury and being killed. However, if there is no predator, remaining provides an advantage, such as allowing continued foraging. We focus on the standard signal detection model and so assume payoffs represent the lifetime change in fitness for each decision outcome (Holen & Sherratt, 2021). In Section 3.7, we alter this assumption to consider how depleting energy reserves affect fleeing (Holen & Sherratt, 2021; McNamara & Trimmer, 2019; Trimmer, Ehlman, McNamara, et al., 2017; Trimmer, Ehlman, & Sih, 2017).

**FIGURE 2:**
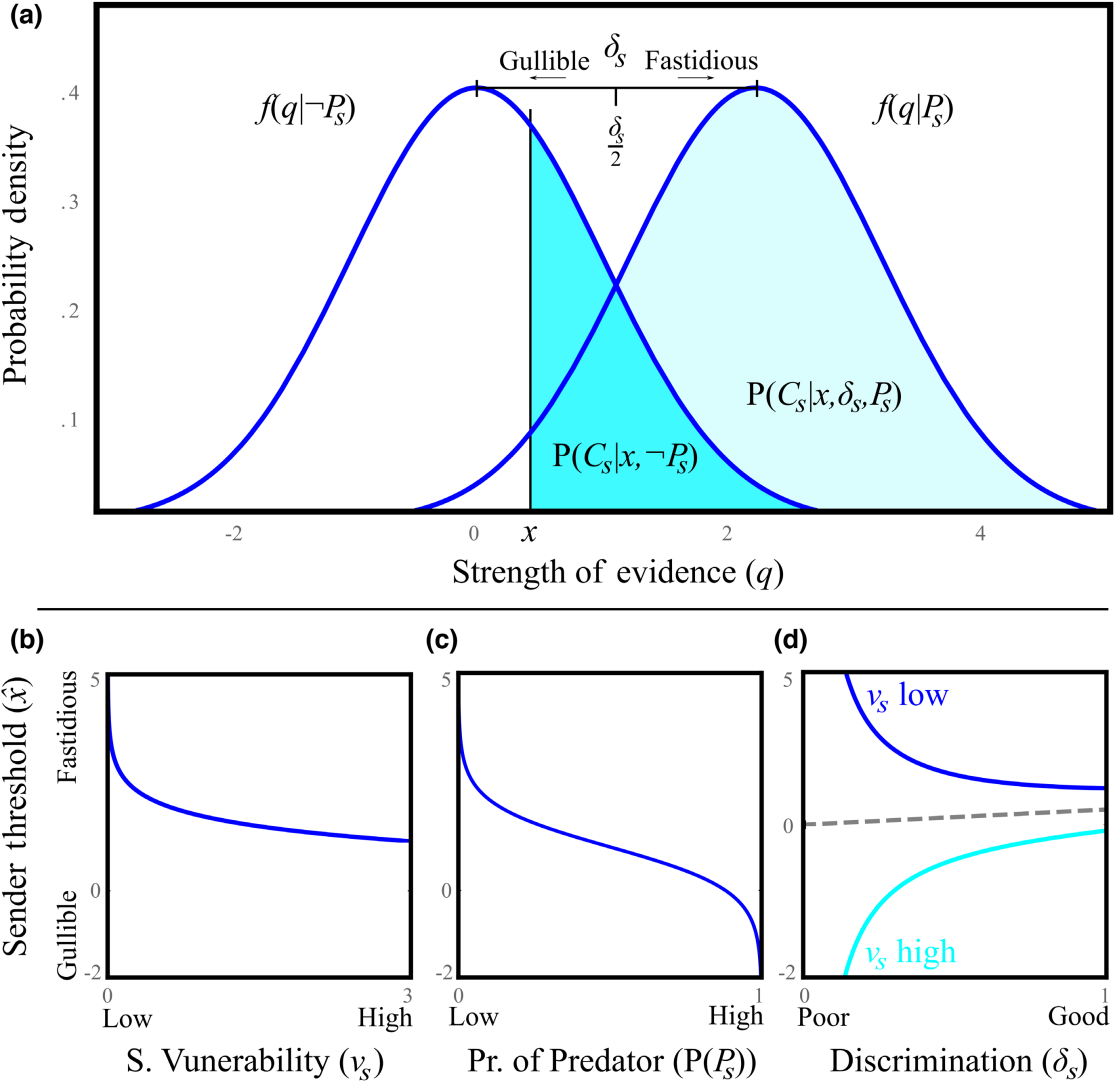
Signal detection of the sender. (a) For any strength of evidence, *q*, there is a chance that it has been produced by the presence of a predator (associated with stronger evidence) or is informational noise. The sender's discrimination, *δ*_*s*_, is the distance between the means of the distribution of an observed level of evidence given no predator (noise distribution) and the distribution when there is a predator (signal and noise distribution). Any detection threshold, *x*, entails a certain probability of correct detections, missed detections, false alarms, and correct rejections. At the point *δ*_*s*_/2, the conditional probability of errors are equal and minimized and adjusting for the baseline probability of predator presence, $\left(1/\delta_{s}\right)\ln\left(P\left(\neg P_{s}\right)/P\left(P_{s}\right)\right)$ (Equation (1a) and (1b)). Thresholds lower than this point are labeled adaptively *gullible*, with those which are higher labeled *fastidious*. (b) Highly vulnerable senders are selected to be more gullible ($P\left(P_{s}\right)=.2$, $\delta_{s}=2$). (c) When the probability of predation is high senders are selected to be more gullible ($\upsilon_{s}=1$, $\delta_{s}=2$). (d) When the sender finds false alarms more costly (dark trendline, $\upsilon_{s}=.5$, $P\left(P_{s}\right)=.5$), poor predator discrimination leads to selection for more fastidious thresholds; however, when missed detections are more costly (light trendline, $\upsilon_{s}=2$, $P\left(P_{s}\right)=.2$), poor discrimination leads to more gullible thresholds; the threshold is selected to be $\delta_{s}/2$ when errors are equally costly (dashed trendline).

According to signal detection theory, evidence gained by an animal's sensory system can lead to two types of correct decisions and two types of incorrect decisions (Figure 2a). Observed sensory evidence varies in strength and can never be perfect, so prey can only set a detection threshold level *x* above which they decide a predator is present and flee. As such, the two types of correct categorization are: (1) *Correct detection*, fleeing from a predator given one is present, which occurs with probability $P\left(C_{s}|P_{s};x,\delta_{s}\right)P\left(P_{s}\right)$, and (2) *correct rejection*, remaining given a predator is absent, $P\left(\neg C_{s}|\neg P_{s};x\right)P\left(\neg P_{s}\right)$. The two types of error decision are: (3) *false alarms*, fleeing given the predator is absent, $P\left(C_{s}|\neg P_{s};x\right)P\left(\neg P_{s}\right)$, and (4) *missed detection* given a predator is present, $P\left(\neg C_{s}|P_{s};x,\delta_{s}\right)P\left(P_{s}\right)$. In setting a threshold, a sender can only trade‐off one kind of error for the other but cannot simultaneously reduce both. Only when discrimination is improved are both types of errors simultaneously reduced.

Assuming the common equal‐variance Gaussian model and utility/fitness function (Green & Swets, 1966; Wickens, 2002) the sender's optimal detection threshold that maximizes the fitness of a detecting sender species is:

(1a) $$\hat{x}=\frac{\delta_{s}}{2}+\frac{1}{\delta_{s}}\ln\left(\frac{P\left(\neg P_{s}\right)}{P\left(P_{s}\right)}\right)+\frac{1}{\delta_{s}}\ln\left(\frac{1}{\upsilon_{s}}\right)$$

(1b) $$\left(\begin{array}{c} \text{Optimal}\\ \text{Sender}\\ \text{Threshold} \end{array}\right)=\left(\begin{array}{c} \mathrm{Opt}.\;\text{Send}.\\ \text{Reliability}\\ \text{Threshold} \end{array}\right)+\left(\begin{array}{c} \text{Adaptive Biasing}\\ \mathrm{due}\;\text{to}\\ \text{Sender Vulnerability} \end{array}\right)$$

Equation (1a) and (1b) reveals how the sender's optimal detection threshold is affected by (1) the payoffs associated with fleeing and remaining, via the summary relative vulnerability parameter, *ν*_*s*_ (see below); (2) the background probability of a predator being present, P(*P*_*s*_); and (3) the sender's discrimination, *δ*_*s*_. Here, *δ*_*s*_ is the ability to discriminate cues for predator presence from noise. A higher *δ*_*s*_ means that there will be a higher probability of the predator being correctly detected for any threshold level, hence better discrimination.

The sender's optimal threshold ($\hat{x}$) is adaptively biased away from the threshold that maximizes sender reliability, due to the relative costs of false alarms compared to missed detections. Sender reliability is formally defined as the combined probability of correct detection and rejection: $P\left(\neg C_{s}|\neg P_{s}\right)P\left(\neg P_{s}\right)+P\left(C_{s}|P_{s}\right)P\left(P_{s}\right)$. So sender reliability is maximized when combined correct categorizations are maximized, which entails that combined errors are minimized. The first two terms of Equation (1a) and (1b) locate this reliability maximizing threshold, which is also the threshold that would make false alarms and missed detections equally probable (Figure 2a). That is, the maximum sender reliability (minimum of combined errors), occurs at the threshold where false alarms and missed detections are equally probable. The third term of Equation (1a) and (1b) gives the *adaptive biasing* of $\hat{x}$ away from this maximum sender reliability threshold. Adaptive biasing makes one type of error less probable than the other, because of the differences in their consequences for fitness. Lower values of $\hat{x}$ are adaptively *gullible* thresholds (Wiley, 2006, 2013, 2015, 2017) and make individuals more responsive to potential threats. Consequently, gullible thresholds mean individuals are more likely to correctly flee if a predator is present, but at the cost of incorrectly fleeing more often (more false alarms). Conversely, higher values of $\hat{x}$ are *fastidious*, so individuals are less responsive to threats (more missed detections) but will unnecessarily flee less.

The degree of adaptive biasing is a consequence of the payoffs associated with fleeing and remaining, along with discrimination. The fitness consequences of a sender's decisions can be summarized by *ν*_*s*_ = *b*_*s*_/*c*_*s*_, which is the ratio of the benefit of avoiding predators due to correct detections, over the cost of unnecessary fleeing due to false alarms. The benefit of avoiding predators, *b*_*s*_, is larger when the risk of predation is high compared to the cost of fleeing. Whereas, the cost of unnecessarily fleeing, *c*_*s*_, is larger when there are substantial benefits to remaining and fleeing is energetically costly. As the risk of predation is often likely to be the most important parameter dictating the behavior of *ν*_*s*_, we label *ν*_*s*_ the *sender's relative vulnerability*. The sender's relative vulnerability increases as a function of *b*_*s*_, so higher predation risk drives larger values of *ν*_*s*_, which leads to a more gullible threshold being selected (Figure 2b). However, when the costs of unnecessary fleeing are large, relative to predation risk, *ν*_*s*_ will be low, selecting for a fastidious threshold. Furthermore, a higher probability of a predator, P(*P*_*s*_), generally selects for a more gullible threshold (Figure 2c; for an exception see Section 3.7). This is because when encounters with a predator are more likely, deciding there is a predator on less evidence is more often correct. Sender's discrimination moderates the effect of adaptive biasing on their optimal detection threshold (Figure 1d). When detection is easy (*δ*_*s*_ is large), senders should set a threshold close to the level that maximizes sender reliability; however, when detection is difficult, errors are more frequent, and more extreme adaptive biasing will be selected to avoid one type of error over the other.

### 2.2 Conditions for selection for eavesdropping

An eavesdropping receiver uses the sender species' call to decide whether or not to flee. We assume a population of senders who call on their optimal threshold. The receiver faces an analogous signal detection problem in detecting a sender's call, as the sender does in detecting a predator. Receivers will vary in their detection threshold *y* for correct call detection, *D*_*r*_, occurring with probability $P\left(D_{r}|y,\delta_{r},C_{s}\right)P\left(C_{s}\right)$; which will also determine the probability of falsely detecting a call, $P\left(D_{r}|y,\neg C_{s}\right)P\left(\neg C_{s}\right)$; correctly rejecting the presence of a call, $P\left(\neg D_{r}|y,\neg C_{s}\right)P\left(\neg C_{s}\right)$; missing a detection of a call, $P\left(\neg D_{r}|y,\delta_{r},C_{s}\right)P\left(C_{s}\right)$. A receiver's call detection also has its own sensitivity index, *δ*_*r*_, which formally defines receiver reception. When reception is better, calls are more often detected when they are present. We assume receivers must trade‐off errors in call reception; however, we are only indirectly interested in the receiver's threshold *y*. Instead, we are primarily interested in when selection will favor receivers who flee given a sender's call (eavesdropping), rather than never flee given a call.

We model the fitness of a receiver by considering the outcomes of eavesdropping, assuming a predator arrives and may be spotted by a sender; the sender may in turn make an alarm call; and the receiver's reception of the call is affected by noise (Figure 1). In particular, the fitness of a receiver is the expected value of each combination of the following events: (1) a predator of the sender is present or absent, (2) a predator of the receiver is present or absent, (3) the sender called or was silent, (4) receiver correctly detected or missed the call, (5) the receiver flees to calls (eavesdropping) or does not. Later, we also assess the influence of (6) whether the receiver personally detects the predator or not. A predator of the receiver can either be shared by the sender or be a unique predator that only threatens the receiver. A receiver's payoffs for risking predation, safely remaining and fleeing may also differ from the senders. Figure 1 depicts the logic of the model; the full expression for receiver fitness is cumbersome and so it is included in the Appendix S1.

Eavesdropping is selected when the benefit gained by the receiver from avoiding their own predators due to detecting calls, is greater than the cost of unnecessarily fleeing when call detections occur in the absence of its predators:

(2a) $$b_{r}P\left(D_{r},P_{r}\right)>c_{r}P\left(D_{r},\neg P_{r}\right)$$

An equivalent way to describe the condition for eavesdropping is in terms of how call detections by the receiver correspond to predators *of the sender*, weighted by the relative vulnerability of the receiver to the sender's predators, *ν*_*r*_.

(2b) $$\upsilon_{r}P\left(D_{r},P_{s}\right)>P\left(D_{r},\neg P_{s}\right)$$

(2c) $$\upsilon_{r}=\frac{b_{r}P\left(P_{r}|P_{s}\right)-c_{r}P\left(\neg P_{r}|P_{s}\right)}{c_{r}P\left(\neg P_{r}|\neg P_{s}\right)-b_{r}P\left(P_{r}|\neg P_{s}\right)}$$

A receiver's detection of a call depends on whether there was a predator of the sender, influencing calling, as well as the receiver's own threshold for call detection. If a predator of the sender is present, a receiver will detect a call if either the sender correctly called and this was correctly perceived, or the sender missed calling, but a call was falsely perceived, $P\left(D_{r}|P_{s}\right)=P\left(D_{r}|C_{s}\right)P\left(C_{s}|P_{s}\right)+P\left(D_{r}|\neg C_{s}\right)P\left(\neg C_{s}|P_{s}\right)$. Similarly, if a predator of the sender is absent, the receiver will still detect a call if there was a sender false alarm that the receiver correctly perceived, or if the receiver falsely perceived a call, $P\left(D_{r}|\neg P_{s}\right)=P\left(D_{r}|\neg C_{s}\right)P\left(\neg C_{s}|\neg P_{s}\right)+P\left(D_{r}|C_{s}\right)P\left(C_{s}|\neg P_{s}\right)$.

For eavesdropping to be selected, call detections must correspond to a threat to the receiver. If a call is perceived, it will warn of a predator of the receiver if either there is a predator shared by the sender or a unique predator of the receiver, $P\left(D_{r},P_{r}\right)=P\left(P_{r}|P_{s}\right)P\left(D_{r},P_{s}\right)+P\left(P_{r}|\neg P_{s}\right)P\left(D_{r},\neg P_{s}\right)$. By fleeing, the receiver will gain the benefit of avoiding this predator, *b*_*r*_. A call may be perceived in absence of a predator of the receiver if either a unique predator of the sender has appeared or there is no predator of either species, $P\left(D_{r},\neg P_{r}\right)=P\left(\neg P_{r}|P_{s}\right)P\left(D_{r},P_{s}\right)+P\left(\neg P_{r}|\neg P_{s}\right)P\left(D_{r},\neg P_{s}\right)$. If the receiver flees, the cost of unnecessarily fleeing to a call will be suffered, *c*_*r*_.

## 3 RESULTS

### 3.1 Threat of predators to receiver

Eavesdropping is selected when the receiver's detection of calls corresponds to a substantial threat, and this will occur if predators of the receiver are more likely to be encountered or are particularly dangerous (Equation (2a) and (2b); for an exception see Section 3.7). This means eavesdropping is favored when the receiver's predators pose a major cost, and unnecessarily fleeing is energetically cheap (linearly increasing with *b*_*r*_ and decreasing with *c*_*r*_). Selection for eavesdropping linearly increases with the probability that a predator of the sender is also shared by the receiver, P(*P*_*r*_|*P*_*s*_). However, selection for eavesdropping also linearly increases with the probability a unique predator of the receiver is present, P(*P*_*r*_|¬*P*_*s*_). This odd situation arises because some calls, which are false alarms from the sender's perspective, will by chance coincide with the presence of a unique predator of the receiver. We are unaware of any reports of coincidence‐based eavesdropping, so focus on eavesdropping resulting from shared predators.

### 3.2 Predator relevance

Eavesdropping is selected if the sender and receiver are vulnerable to similar predators, so that calls have relevance. We formally examine relevance by comparing how the relative vulnerabilities of the sender (*ν*_*s*_) and receiver (*ν*_*r*_), to the predators of the sender, affects selection for eavesdropping. Recall, the relative vulnerability is the predation risk from a predator compared to the benefits of remaining in place, both adjusted for the cost of fleeing (Table 1). In the case of the receiver, it is also adjusted for predator overlap (Equation 2c).

**FIGURE 3:**
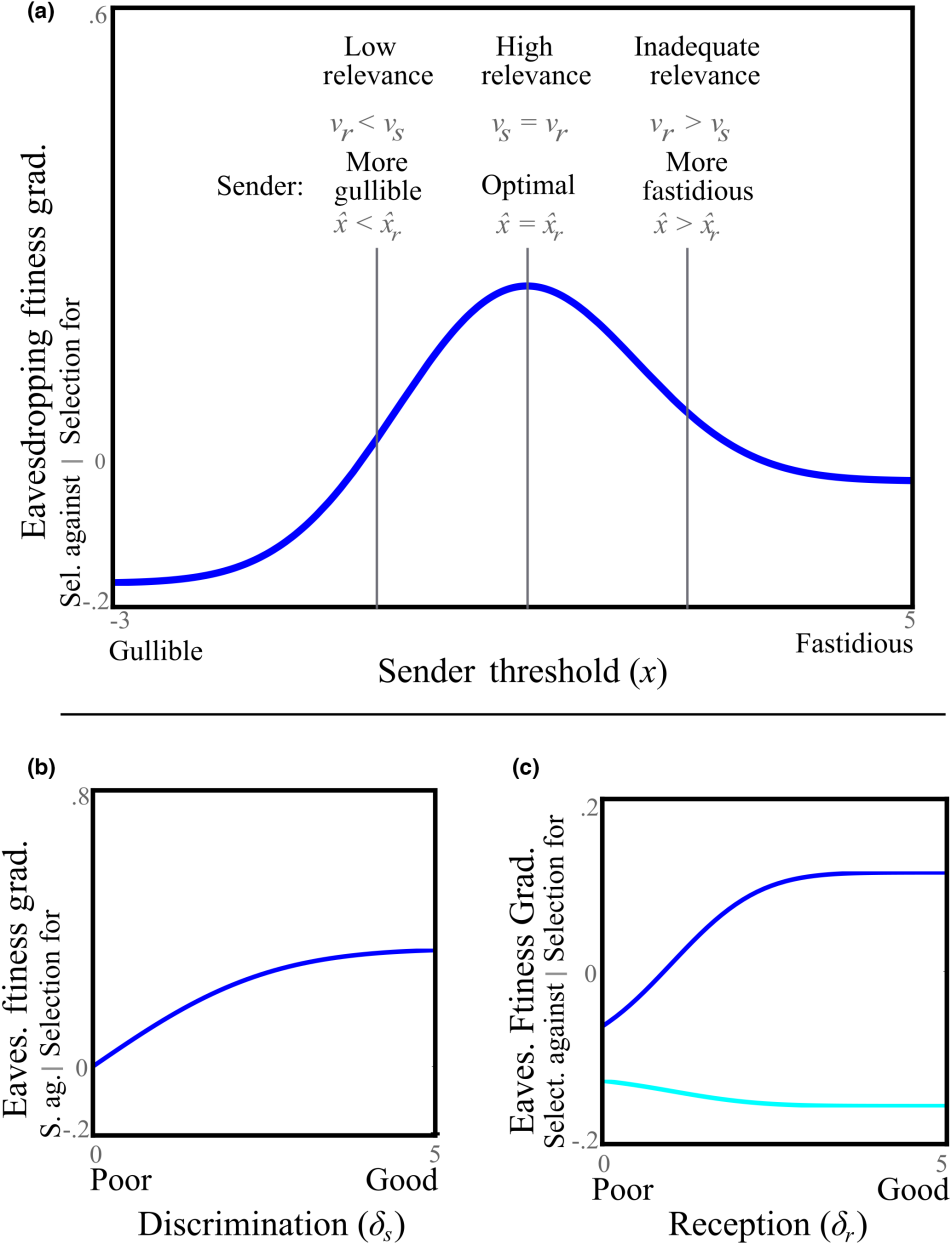
The fitness gradient of eavesdropping versus sender threshold, discrimination, and reception. When the fitness gradient of eavesdropping is positive, eavesdropping (responding to a detected call by the receiver) is selected for. (a) Selection for eavesdropping as a result of biasing of the sender's threshold ($P\left(P_{s}\right)=.2$, y=1, $\delta_{s}=2$, $\delta_{r}=2$, $\upsilon_{r}=3$). Lines are examples of senders with thresholds that are (1) more gullible than is optimal, (2) optimal, and (3) more fastidious than is optimal, for eavesdropping. Selection is maximized when the sender's threshold, $\hat{x}$, is equivalent to the threshold that maximizes selection for eavesdropping, ${\hat{x}}_{r}$, occurring when there is high relevance ($\upsilon_{r}=\upsilon_{s}$). If there is low relevance ($\upsilon_{r}<\upsilon_{s}$), then the sender is more gullible than is optimal for the receiver ($\hat{x}<{\hat{x}}_{r}$). If there is inadequate relevance ($\upsilon_{r}>\upsilon_{s}$), the sender is more fastidious than is optimal for the receiver ($\hat{x}>{\hat{x}}_{r}$). (b) In general, better discrimination leads to stronger selection for eavesdropping ($P\left(P_{s}\right)=.2$, y=1, $\delta_{r}=2$, $\upsilon_{s}=1$, $\upsilon_{r}=1$). (c) Better reception is associated with stronger selection for eavesdropping when other conditions are favorable (dark trendline) ($P\left(P_{s}\right)=.2$, y=1, $\delta_{s}=2$, $\upsilon_{s}=1$, $\upsilon_{r}=1$), but selects against it when they are unfavorable (light trendline) ($\upsilon_{r}=.01$).

Receivers benefit most from eavesdropping if they have similar vulnerability to senders, so calling sufficiently balances missed detection and false alarms in a way that is favorable for the eavesdropper (Figure 3a). In particular, the sender's threshold, *x*, that maximally selects for eavesdropping is:

(3a) $${\hat{x}}_{r}=\frac{\delta_{s}}{2}+\frac{1}{\delta_{s}}\ln\left(\frac{P\left(\neg P_{s}\right)}{P\left(P_{s}\right)}\right)+\frac{1}{\delta_{s}}\ln\left(\frac{1}{\upsilon_{r}}\right)$$

(3b) $$\left(\begin{array}{c} \text{Optimal}\\ \text{Send}.\mathrm{Thr}.\\ \text{for Receiver} \end{array}\right)=\left(\begin{array}{c} \mathrm{Opt}.\;\text{Send}.\\ \text{Reliability}\\ \text{Threshold} \end{array}\right)+\left(\begin{array}{c} \text{Adaptive Biasing}\\ \mathrm{due}\;\text{to}\\ \text{Receiver Vulnerability} \end{array}\right)$$

Note, ${\hat{x}}_{r}$ is nearly identical in form to the threshold which maximizes sender's fitness, $\hat{x}$ (Equation (1a) and (1b)), except for the receiver's relative vulnerability appearing in the place of the sender's. This confirms that high relevance selects for eavesdropping, because eavesdropping is maximized when the sender calls on the same threshold that they would if sender and receiver had equivalent relative vulnerabilities (if $\upsilon_{r}=\upsilon_{s}$, then $\hat{x}={\hat{x}}_{r}$). In general, perfectly high relevance, $\upsilon_{r}=\upsilon_{s}$, will occur when all predators are shared, with there being no unique predators, and costs and benefits are the same. If receivers are less vulnerable than senders there is *low relevance* ($\upsilon_{r}<\upsilon_{s}$), and senders will call on a threshold that is more gullible than is ideal for the receiver ($\hat{x}<{\hat{x}}_{r}$). Low relevance implies the sender produces too many false alarms from the perspective of the receiver. However, if a receiver is more vulnerable than the sender, $\upsilon_{r}>\upsilon_{s}$, the sender will be too fastidious in calling, missing more instances of predator presence than is ideal for the receiver ($\hat{x}>{\hat{x}}_{r}$). In this case, calls have *inadequate relevance*, selecting against eavesdropping because they do not adequately reduce predation for the receiver, due to missing too many detections.

### 3.3 Sender discrimination

Superior sender's discrimination, *δ*_*s*_, generally selects for eavesdropping (Figure 3b). This is because senders with superior discrimination will have a greater probability of correctly detecting shared predators and fewer missed detections, no matter the sender's threshold (making P(*C*_*s*_|*P*_*s*_) large). Consequently, the receiver is more likely to benefit by avoiding shared predators (for unusual exceptions see Appendix S1).

### 3.4 Receiver reception

Superior reception can either increase or decrease the benefits of eavesdropping (Figure 3c). As both correct and false alarm calls are detected at higher rates when reception is good, whether better reception selects for eavesdropping depends on if the fitness gained by detecting more correct calls outweighs that lost due to perceiving more false alarm calls. In particular, reception increases selection for eavesdropping when $b_{r}P\left(C_{s},P_{r}\right)>c_{r}P\left(C_{s},\neg P_{r}\right)$. This shows that, for improved reception to favor eavesdropping, calls must already correspond to a dangerous predator of the receiver.

### 3.5 Receiver alarm call detection threshold

Analogous to the sender's detection threshold, eavesdropping is most strongly selected at intermediate values of receiver detection thresholds, *y*, which are biased to manage the trade‐off between false and missed perceptions. If the receiver often encounters dangerous predators, more gullible receiving thresholds that prioritize avoiding encounters will select for eavesdropping. However, if the receiver rarely risks predation, then more fastidious thresholds will select for eavesdropping. When the receiver's reception is better, biasing of the call detection threshold is less important in selection for eavesdropping, because errors are rare.

### 3.6 Personal information

If we assume the receiver can directly detect the predator, using personal information, the condition for selection for eavesdropping becomes:

(4) $$b_{r}P\left(D_{r},P_{r}\right)\left(1-P\left(D_{p}|P_{r}\right)\right)>c_{r}P\left(D_{r},\neg P_{r}\right)\left(1-P\left(D_{p}|\neg P_{r}\right)\right)$$

Here, $P\left(D_{p}|P_{r}\right)$ is the probability a receiver correctly detects one of their own predators using personal information, with $P\left(D_{p}|\neg P_{r}\right)$ being the probability the receiver falsely detects one of their own predators. It can be seen that when personal discrimination of predators is poor, so there are many missed detections using personal information, eavesdropping is most strongly favored; that is, $1-P\left(D_{p}|P_{r}\right)\to 1$, as personal discrimination becomes poorer. Whereas, if a receiver is good at personally discriminating predators, then eavesdropping will be selected against, because threats are often identified without recourse to eavesdropping.

### 3.7 Depleted reserves caused by repeated fleeing

So far, we have followed the standard model and assumed payoffs represent the lifetime change in fitness of different outcomes. This also means that payoff parameters are functionally unrelated, and we have examined each payoff's effect on eavesdropping by varying it freely. However, when predators are very common, prey may deplete their energy reserves by fleeing too frequently, so eventually it becomes favorable to continue foraging and replenish reserves (Holen & Sherratt, 2021; McNamara & Trimmer, 2019; Trimmer, Ehlman, McNamara, et al., 2017; Trimmer, Ehlman, & Sih, 2017).

**FIGURE 4:**
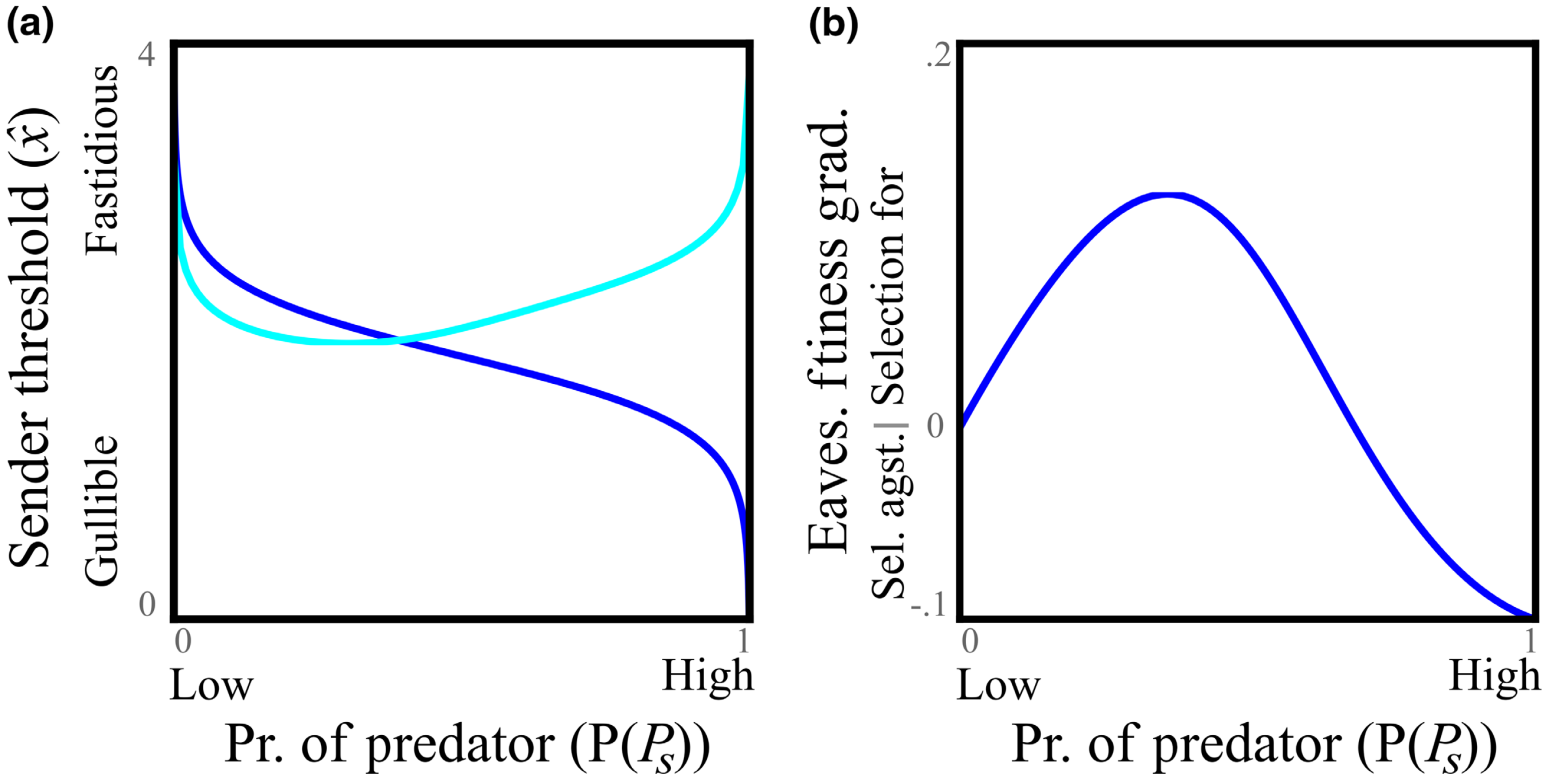
Sender threshold and selection on eavesdropping assuming reserves are depleted by fleeing. (a) Optimal sender threshold: standard model is dark blue trendline, energy reserve model is light blue trendline ($\delta_{s}=2$, $\upsilon_{s}=2$, $d_{s}=.05$). (b) Selection for eavesdropping decreases the high probability of predator presence, but increases at moderate probability of predator presence ($\delta_{s}=8$, $\delta_{r}\to \infty$, $d_{s}=d_{r}=.05$, $\alpha_{s}=\alpha_{r}=1$, $P\left(P_{r}|P_{s}\right)=.9$, $P\left(P_{r}|\neg P_{s}\right)=.2$; see Appendix S1 for explanation of parameters).

Energy reserve models predict that a high and ongoing probability of encountering predators leads to prey being bolder, to avoid depleting their energy reserves. These models assume a dependency between the payoff of fleeing and predator presence, which is not assumed in the standard model. The fitness of the sender under repeated fleeing can be derived by making the following assumptions (see McNamara & Trimmer, 2019, for a full description). First, a sender who remains when a predator is absent gains payoff *α*_*s*_, and remaining when a predator always leads to death and a payoff of 0. Secondly, sender regains a unit of energy reserves with probability *θ*_*s*_. If prey flees, they gain *α*_*s*_ only under the condition that they replenish unit of reserves lost fleeing, and then accrue a further unit of reserves, so that the total payoff of fleeing is $\alpha_{s}\theta_{s}^{2}$. Here, the probability of regaining reserves is a decreasing function of predator presence: $\theta_{s}\left(P\left(P_{s}\right)\right)$ is decreasing. These assumptions lead to a reversal of predictions such that at high continued probability of encountering a predator, senders become more fastidious rather than gullible (Figure 4a; McNamara & Trimmer, 2019).

When repeated encounters deplete reserves, a high probability of encountering predators selects against using heterospecific alarm calls. We assumed the receiver's fitness has an analogous form to the sender, which depends on the probability that the receiver is able to regain lost energy, after fleeing due to eavesdropping $\theta_{r}$. A high probability of encountering a predator leads to selection against eavesdropping, rather than increased eavesdropping as in the standard model (Figure 4b). This is analogous to the finding that senders become more fastidious in calling; here, receivers become less likely to flee in response to calls. As with the sender, this result occurs because increasing the prevalence of predators has two countering effects on eavesdropping. First, it increases the level of threat (selecting for eavesdropping); this causes selection for eavesdropping at moderate probability of encountering a predator. Secondly, it means that energy will be lost because of fleeing (selecting against eavesdropping). The second effect dominates when there are very high levels of predation risk.

### 3.8 Reliability

Previous definitions state that high eavesdropping reliability entails both few false alarms and few missed detections, from the perspective of the receiver, but have not formally characterized reliability in eavesdropping (Magrath et al., 2015; Searcy & Nowicki, 2005). We define *eavesdropping reliability*, ρ, as the total probability of correct categorization, which is the probability of a correct detection of a predator of the receiver, plus the probability of a correct rejection. This is analogous to sender reliability but defined with respect to predators of the receiver rather than sender. A receiver correct detection occurs if the sender correctly calls to a shared predator (also a sender correct detection), or if a call is given to a unique predator of the receiver (a sender false alarm): $P\left(C_{s},P_{r}\right)=P\left(P_{r}|P_{s}\right)P\left(C_{s}|P_{s}\right)P\left(P_{s}\right)+P\left(P_{r}|\neg P_{s}\right)P\left(C_{s}|\neg P_{s}\right)P\left(\neg P_{s}\right)$. A receiver correct rejection occurs if either the sender correctly rejects the presence of a non‐predator of both species (also a sender correct rejection) or misses the detection of a predator that only threatens the sender (a sender missed detection): $P\left(\neg C_{s},\neg P_{r}\right)=P\left(\neg P_{r}|\neg P_{s}\right)P\left(\neg C_{s}|\neg P_{s}\right)P\left(\neg P_{s}\right)+P\left(\neg P_{r}|P_{s}\right)P\left(\neg C_{s}|P_{s}\right)P\left(P_{s}\right)$.

Senders whose calls are highly relevant are more favorable for eavesdropping than those which maximize only eavesdropping reliability. The sender's threshold that maximizes eavesdropping reliability, ${\hat{x}}_{\rho }$, is given by the threshold that maximizes sender reliability (first two terms Equation (5a) and (5b)), plus a term that adjusts the threshold depending on the overlap in predators (third term Equation (5a) and (5b)):

(5a) $${\hat{x}}_{\rho }=\frac{\delta_{s}}{2}+\frac{1}{\delta_{s}}\ln\left(\frac{P\left(\neg P_{s}\right)}{P\left(P_{s}\right)}\right)+\frac{1}{\delta_{s}}\ln\left(\frac{P\left(\neg P_{r}|\neg P_{s}\right)-P\left(P_{r}|\neg P_{s}\right)}{P\left(P_{r}|P_{s}\right)-P\left(\neg P_{r}|P_{s}\right)}\right)$$

(5b) $$\left(\begin{array}{c} \text{Optimal}\\ \text{Send}.\mathrm{Thr}.\\ \text{Eaves}.\;\text{Reliability} \end{array}\right)=\left(\begin{array}{c} \mathrm{Opt}.\;\text{Send}.\\ \text{Reliability}\\ \text{Threshold} \end{array}\right)+\left(\begin{array}{c} \text{Biasing}\;\mathrm{due}\;\text{to}\\ \text{Differences in}\\ \mathrm{Set}\;\text{of Predators} \end{array}\right)$$

In particular, to produce more receiver correct categorizations, the sender must set a more gullible threshold when there are more encounters with predators of the receiver (whether shared or unique), while being more fastidious if there are fewer encounters with predators of the receiver. To allow easier comparison, the threshold that maximizes selection for eavesdropping (not just reliability) can be rewritten:

(3c) $${\hat{x}}_{r}=\frac{\delta_{s}}{2}+\frac{1}{\delta_{s}}\ln\left(\frac{P\left(\neg P_{s}\right)}{P\left(P_{s}\right)}\right)+\frac{1}{\delta_{s}}\ln\left(\frac{c_{r}P\left(\neg P_{r}|\neg P_{s}\right)-b_{r}P\left(P_{r}|\neg P_{s}\right)}{b_{r}P\left(P_{r}|P_{s}\right)-c_{r}P\left(\neg P_{r}|P_{s}\right)}\right)$$

While similar in form, the selection maximizing threshold accounts for not only the probability of correct categorizations from the receiver's perspective, but also the fitness gained or lost from decisions made as a consequence of these categorizations (*b*_*r*_, *c*_*r*_). This means that calls low in eavesdropping reliability can still be favorable, if the receiver experiences large benefits from avoiding predation or unnecessary fleeing. In other words, high call relevance, entailing both a similar set of predators and fleeing decision consequences between sender and receiver, is most advantageous for eavesdropping.

To fully assess how reliable a sender is for eavesdropping it is necessary to measure both when a sender's calls and omissions correspond to a predator of a receiver, as well as when they do not. Table 3 describes how to calculate the different quantities related to reliability (Kohl, 2012, gives calculations generally). Previous work has tested the hypothesis that eavesdropping is more likely to be favored when a large proportion of calls are given to a predator of the receiver, with few being receiver false alarms, by measuring the number of calls in the presence and absence of a predator (Magrath et al., 2009; Meise et al., 2018). This quantity is the probability of a predator of the receiver given a call (Goodale et al., 2010), and measures how informative a call is about the presence of a predator. Another common measure, capturing how often calls correspond to predators compared with missed detections is the *sensitivity* of a call (Goodale et al., 2010). However, it is also necessary to capture the rate of correct rejections; this can be summarized by the *specificity* of the call, or the degree to which silence is indicative of predator absence. Note, to fully empirically measure reliability a researcher must quantify the number of calls and omissions, both when a predator is present and absent. Reliability is calculated as the number of correct calls and omissions corresponding to predator absence, over the total number of calls and omissions.

**TABLE 3:** Calculating reliability from raw counts of instances of calls or no calls, in the presence or absence of a predator.

Quantity	Calculation
Eavesdropping reliability *ρ*	$\frac{n\left(C_{s},P_{r}\right)+n\left(\neg C_{s},\neg P_{r}\right)}{n\left(C_{s},P_{r}\right)+n\left(C_{s},\neg P_{r}\right)+n\left(\neg C_{s},\neg P_{r}\right)+n\left(\neg C_{s},P_{r}\right)}$
Probability of Predator of Receiver given Call $P\left(P_{r}|C_{s}\right)$	$\frac{n\left(C_{s},P_{r}\right)}{n\left(C_{s},P_{r}\right)+n\left(C_{s},\neg P_{r}\right)}$
Sensitivity of Calls for Receiver $P\left(C_{s}|P_{r}\right)$	$\frac{n\left(C_{s},P_{r}\right)}{n\left(C_{s},P_{r}\right)+n\left(\neg C_{s},P_{r}\right)}$
Specificity of Calls for Receiver $P\left(\neg C_{s}|\neg P_{r}\right)$	$\frac{n\left(\neg C_{s},\neg P_{r}\right)}{n\left(\neg C_{s},\neg P_{r}\right)+n\left(C_{s},\neg P_{r}\right)}$
Probability of a Call $P\left(C_{s}\right)$	$\frac{n\left(C_{s}\right)}{n\left(C_{s}\right)+n\left(\neg C_{s}\right)}$
Probability of a Predator of the Receiver $P\left(P_{r}\right)$	$\frac{n\left(P_{r},P_{s}\right)+n\left(P_{r},\neg P_{s}\right)}{n\left(P_{r},P_{s}\right)+n\left(P_{r},\neg P_{s}\right)+n\left(\neg P_{r},\neg P_{s}\right)+n\left(\neg P_{r},\neg P_{s}\right)}$

*Note*: *n* is a function that counts the number of events. For instance, $n\left(C_{s},\neg P_{r}\right)$ is a count of the number of instances of calls when a predator of the receiver was not present. Equivalent quantities for the sender can by substituting instances with predators of the receiver for those with predators of the sender.

## 4 DISCUSSION

We produced a model, based on signal detection theory, to give conditions for the evolution of interceptive eavesdropping and formalize previous language‐based theory (Goodale et al., 2010; Magrath et al., 2015; Schmidt et al., 2010; Seppänen et al., 2007). Eavesdropping is selected when the likely benefit gained by using calls to avoid predators is greater than the cost due to unnecessary fleeing when predators are absent. We found that selection for eavesdropping is maximized when the sender and receiver are ecologically similar and so face the same level of threat from identical predators (Goodale et al., 2010; Seppänen et al., 2007). This is because ecologically similar senders will give calls on a threshold that is biased to trade‐off errors in a way that is also optimal for the receiver. While this biased threshold increases call relevance, it will also increase the total number of calling errors; unexpectedly, this means that ecological similarity of species can override selection resulting from call reliability. In addition, eavesdropping is more strongly selected when there is little noise affecting discrimination of information from the sender, and when there is reduced personal information. However, improved reception of calls can sometimes select against eavesdropping, if it only means more costly false alarms are perceived.

### 4.1 Ecological similarity supports eavesdropping

Ecological similarity of sender and receiver species selects for eavesdropping because of the inherent trade‐off between false alarms and missed detection. It is advantageous for prey to miss fewer predator detections when the risk of predation is higher, yet have fewer false alarms when predation risk is lower. The sender may be adaptively biased to be either (1) more gullible and miss fewer detections, or (2) more fastidious and have fewer false alarms, than is optimal from the receiver's perspective. Eavesdropping is favored most strongly when the sender happens to trade‐off avoiding predation and unnecessarily fleeing in a way that favors the receiver. This occurs when there is high relevance, such that senders and receivers face the same level of threat from identical predators. Indeed, both receiver vulnerability and ecological similarity have been recently shown to be simultaneous predictors of eavesdropping (Meise et al., 2018). Our model predicts that ecological similarity is essential for eavesdropping whenever receivers face low absolute levels of vulnerability. We have demonstrated that it is the advantageous balancing of errors and correct categorizations by the sender that explains why ecological similarity selects for eavesdropping.

Missed detections can select against eavesdropping, even if there are few false alarms. Previous research has emphasized that too many false alarms from the receiver's perspective selects against eavesdropping (Magrath et al., 2009; Meise et al., 2018; Munoz et al., 2015; Palmer & Gross, 2018; Rainey et al., 2004a, 2004b). In such cases, the sender is more vulnerable than the receiver, so they often falsely alarm, producing calls with low relevance. Our model makes clear that missed detections from the receiver's perspective can also select against eavesdropping. This can occur when the receiver is more vulnerable than the sender. Here the sender calls too fastidiously, giving inadequate information about the presence of predators, such that the receiver does not gain enough of an upside of eavesdropping in terms of avoiding predators (calls have inadequate relevance). Therefore, our results fit well with research that emphasizes eavesdropping is selected when calls of a heterospecific effectively stands in for conspecific or personally acquired information (Goodale et al., 2010; Meise et al., 2018; Seppänen et al., 2007).

Rates of false alarms within alarm calling may sometimes be lower than previously assumed. It is often suggested that there will be a large proportion of false alarms, because the cost of predation greatly outstrips the cost of unnecessary fleeing (Magrath et al., 2015; Munn, 1986; Searcy & Nowicki, 2005). This expectation has been supported in some cases: roosters give aerial alarms to a variety of birds in flight, most of which do not pose a direct threat (Gyger et al., 1987). However, our model predicts that because species face gradations of threat, there should be gradations in the rates of false alarms, as well as toleration of false alarms by potential eavesdroppers. Studies comparing a wide variety of species have shown there can be substantial variation in call frequency between species correlated with their vulnerability (Meise et al., 2018), which suggests that there may not always be high rates of false alarms.

### 4.2 Relevance trumps reliability in selecting for eavesdropping

Reliability is less important for eavesdropping than relevance. Sender reliability is formally defined as the probability the sender makes correct decisions—calling when a predator is present and not calling when there is no predator. Eavesdropping reliability is analogous to sender reliability but is defined as the total probability of a sender's correct categorization of predators *from the perspective of the receiver*. Calls high in eavesdropping reliability are more favorable for eavesdropping than those high in sender reliability, because they correctly categorize receiver threats rather than sender threats, which differ depending on the degree of predator overlap. However, calls with high eavesdropping reliability are less favorable for eavesdropping than those with high relevance. That is, assuming equivalent discrimination abilities, senders who minimize the combined total of false alarms and missed detection (high reliability), are less favorable than those who are biased to avoid the type of error that is costlier to the receiver (high relevance). Our model shows the importance of both calling and not calling, whereas empirical research has assessed the effect of reliability by measuring the proportion of calls that are false alarms compared with correct detections (Magrath et al., 2009; Meise et al., 2018). We show that a full assessment of reliability should also count correct rejections and missed detections (Section: *Reliability*). While this may be difficult, the data are needed to calculate both the proportion of instances a predator is present for which a call is given (sensitivity), and the proportion of instances a predator is absent that no call is given (specificity), which together define reliability (Table 3). One option for empirical testing is to observe many equivalent periods of time, and score whether each period has a call or predator, or their absence. However, our model further suggests that to most effectively predict eavesdropping, it is crucial to collect data on the level of vulnerability and energetic expenditure of fleeing for both sender and receiver (assessing relevance).

### 4.3 Informational noise and eavesdropping

Good discrimination of a predator by the sender generally favors eavesdropping. Improved discrimination is the only way by which the probability of false alarms and missed detections can be simultaneously decreased. Our results confirm that this is because a sender with better discrimination can more often detect a predator that is present and miss few detections. This finding helps to explain previous research that has noted that eavesdropping networks form around *sentinel species* with superior vantage points or sensory systems who can more easily discriminate predators (Goodale et al., 2010; Martínez et al., 2018; Martínez & Zenil, 2012; Ridley et al., 2014; Sridhar et al., 2009).

Good reception does not always lead to greater selection for eavesdropping. This is because being better able to perceive calls is not an advantage if those calls are false alarms from the perspective of the receiver. Therefore, the sender's calling must already be effective at distinguishing predators of the receiver for reception to select for eavesdropping. However, when heterospecific alarm calls are relevant, better reception should coevolve with eavesdropping. In contrast to communication with conspecifics, eavesdropping on heterospecific calls may entail perceiving distant, degraded calls, from unpredictable directions, and individuals may lack species‐specific perceptual adaptation to detect call features (Magrath et al., 2015; Murray & Magrath, 2015; Seppänen et al., 2007). As a result, there can be poorer reception of heterospecific than conspecific calls (Grade & Sieving, 2016; Morris‐Drake et al., 2017; Murray & Magrath, 2015). However, we are unaware of any study showing adaptations specifically to perceive heterospecific calls. Furthermore, analogous to a sender detecting a predator, the receiver's threshold for deciding whether there is an alarm call must manage error trade‐offs; gullible receivers are selected to eavesdrop if they face a high predation risk, while fastidious receivers are selected if unnecessarily fleeing is costly.

### 4.4 Predator danger and eavesdropping

All things being equal, there is stronger selection for eavesdropping when predators of the receiver are more prevalent or dangerous. This is because calls are more likely to correspond to a threat, regardless of whether predators are shared with the sender or unique to the receiver. If the predators are shared, then the sender's correct alarm calls will indicate danger. Surprisingly, our model suggests that eavesdropping could even be selected when there are no shared predators, because the sender's false alarm calls can coincide with unique predators of the receiver. This coincidence‐based form of eavesdropping has not been described in nature but could apply to situations where similar‐looking predators nonetheless eat different prey. In this case, false alarms by one species may correspond to real threats to another.

Our model showed that the loss of energy due to repeated fleeing can select against fleeing to heterospecific calls, so that there is a reversal of the prediction that high predation risk leads to eavesdropping, when predator encounters are very frequently and chronically. Broadly, to understand when eavesdropping will evolve, the deadliness of predators must be considered alongside energy production through foraging, and loss through fleeing. Our findings fit with recent theory suggesting prey become bolder and prioritize conserving reserves when predators are very common for extended periods of time (Holen & Sherratt, 2021; McNamara & Trimmer, 2019; Trimmer, Ehlman, McNamara, et al., 2017; Trimmer, Ehlman, & Sih, 2017). In nature, prey may monitor if they are in a period of continued predation, as well as their own reserve levels, to flexibly adjust their use of cues for fleeing (Trimmer, Ehlman, McNamara, et al., 2017; Trimmer, Ehlman, & Sih, 2017).

### 4.5 Poor personal information supports eavesdropping

Consistent with empirical studies, our model finds that restricted personal information favors eavesdropping. This is because, if personal information is already sufficiently successful in allowing detecting predators, there is no need to rely on eavesdropping. Therefore, we confirm the logic of previous studies of eavesdropping in natural communities, which have found that species that forage in niches that restrict their ability to detect predators are more likely to eavesdrop on heterospecific alarm calls than those species that have a clear view and can rely on personal information (Jones & Sieving, 2019; Martínez & Zenil, 2012). Furthermore, experiments have shown the same pattern occurs within species. New Holland honeyeaters (*Philodonyris novaehollandiae*) responded to alarm calls more strongly when they are feeding on flowers compared with perched in the open hawking for insects; and Australian magpies (*Cracticus tibicen*) respond to alarm calls more strongly when a visual barrier experimentally blocked personal information (McLachlan et al., 2019; Ratnayake et al., 2021).

### 4.6 Eavesdropping and group dynamics

Our model provides a foundation for future modeling to examine how eavesdropping can lead to communication and deception. We assumed that eavesdropping did not affect the fitness of the sender, which appears to be common in natural communities (Peake, 2005). However, eavesdropping can set the stage for communication and deception (Magrath et al., 2020). Some species produce alarm calls to warn heterospecifics, even about predators to which the sender is not vulnerable (Flower, 2011; Munn, 1986; Radford et al., 2011; Rasa, 1983; Ridley et al., 2007; Ridley & Raihani, 2006). These signaling systems can evolve for mutualistic defense against predators (Rasa, 1983), or sender and receivers may gain different advantages (Flower, 2011; Munn, 1986; Radford et al., 2011; Ridley & Raihani, 2006). An instance of the latter involves pied babblers who improve their defense against predators by responding to drongo (*Dicrurus adsimilis*) alarm calls but must tolerate deceptive false alarms produced by drongos to steal the babbler's food (Flower, 2011; Radford et al., 2011; Ridley & Raihani, 2006). Even in the absence of heterospecific signaling, eavesdropping may confer benefits to senders by having more fleeing animals nearby that confuse a predator or discourage a predator from persisting with hunting (Charnov, 1976; Goodale et al., 2019; Maynard Smith, 1965). Our model, by dealing with the scenario of no fitness effect on the sender, provides a basis for elaboration to further explore these and other complex interactions among species.

## AUTHOR CONTRIBUTIONS

**Cameron Rouse Turner:** Conceptualization (lead); formal analysis (lead); writing – original draft (lead). **Matt Spike:** Conceptualization (supporting); supervision (supporting). **Robert D. Magrath:** Conceptualization (supporting); resources (lead); supervision (lead); writing – original draft (supporting); writing – review and editing (supporting).

## CONFLICT OF INTEREST STATEMENT

None.

## FUNDING INFORMATION

This research was funded by PhD research grants to Cameron Rouse Turner from the Deutsche Akademische Austauschdienst (DAAD) and the Department of Education, Australian Government.

## Supporting information


Appendix S1

## Data Availability

Data sharing not applicable to this article as no datasets were generated or analysed during the current study.
