# Supplementary material for: The evolution of eavesdropping on heterospecific alarm calls: Relevance, reliability, and personal information
Source: Ecol Evol. 2023 Jul 12;13(7):e10272. doi: 10.1002/ece3.10272 (PMC10337016; doi:10.1002/ece3.10272)
Supplement: Supplementary file 1 — Appendix S1 [file ECE3-13-e10272-s001.docx]

#

**The Evolution of Eavesdropping on Heterospecific Alarm Calls: Relevance, Reliability, and Personal Information**

**Supplementary Material**

1. **FURTHER INFORMATION: SELECTION FOR CALLING DECISIONS BY SENDERS**

We define the fitness payoffs associated with the decision to flee or remain in place in Table S1.

**TABLE S1** Payoffs for fleeing or remaining in the presence or absence of a predator.

|  | *Flee* | *Remain* |
| --- | --- | --- |
| *No Predator* | [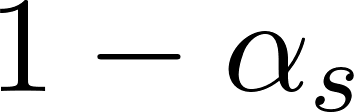](https://www.codecogs.com/eqnedit.php?latex=1-%5Calpha_s#0) | [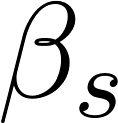](https://www.codecogs.com/eqnedit.php?latex=%5Cbeta_s#0) |
| *Predator* | [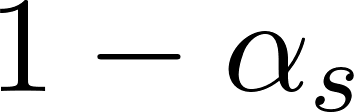](https://www.codecogs.com/eqnedit.php?latex=1-%5Calpha_s#0) | [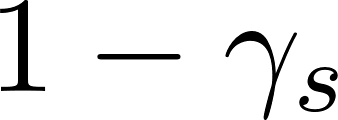](https://www.codecogs.com/eqnedit.php?latex=1-%5Cgamma_s#0) |

First, [
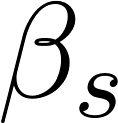
](https://www.codecogs.com/eqnedit.php?latex=%5Cbeta_s#0) increases with the value of foraging and other opportunities resulting from avoiding unnecessary fleeing. Secondly, [
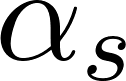
](https://www.codecogs.com/eqnedit.php?latex=%5Calpha_s#0) increases with higher energetic costs involved with fleeing. Finally, [
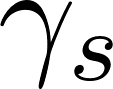
](https://www.codecogs.com/eqnedit.php?latex=%5Cgamma_s#0) is the cost associated with remaining when a predator is present, and increases with predation risk. The payoff of safely remaining is larger than that of fleeing, which is larger than risking predation,[
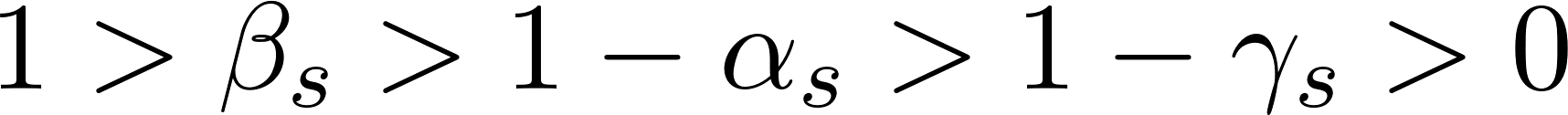
](https://www.codecogs.com/eqnedit.php?latex=1%3E%5Cbeta_s%20%3E%201-%5Calpha_s%20%3E%201-%5Cgamma_s%3E0#0).

In the main text, we summarise the basic payoffs into meaningful higher-level parameters. First, the net fitness benefit obtained by fleeing to avoid predation is [
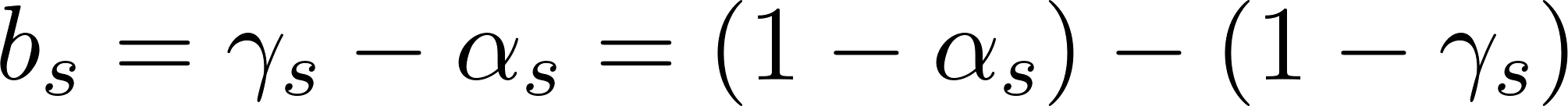
](https://www.codecogs.com/eqnedit.php?latex=b_s%20%3D%20%5Cgamma_s%20-%20%5Calpha_s%20%3D%20(1-%5Calpha_s)%20-%20(1-%5Cgamma_s)%20#0). Secondly, the net cost of unnecessary fleeing is [
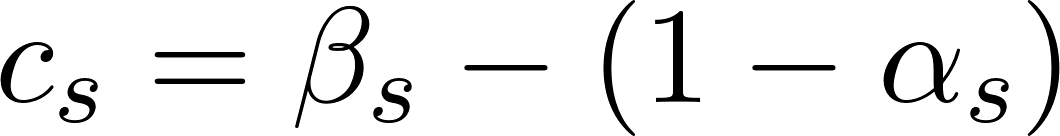
](https://www.codecogs.com/eqnedit.php?latex=c_s%20%3D%20%5Cbeta_s%20-%20(1-%5Calpha_s)#0). Finally, the relative vulnerability of the sender to its predators is [
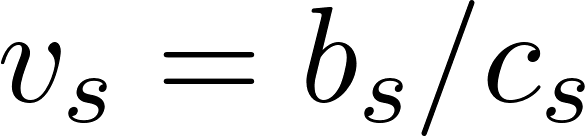
](https://www.codecogs.com/eqnedit.php?latex=v_s%20%3D%20b_s%20%2Fc_s#0). We suggest the cost of predation is the key payoff driving differences in calling behaviour, although it is scaled relative to the benefit of safely remaining and cost of fleeing. So we predominantly focus on [
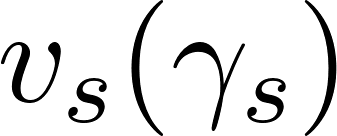
](https://www.codecogs.com/eqnedit.php?latex=v_s(%5Cgamma_s)#0), and in particular focus on how increases in [
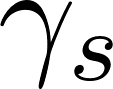
](https://www.codecogs.com/eqnedit.php?latex=%5Cgamma_s#0) (and therefore [
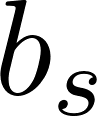
](https://www.codecogs.com/eqnedit.php?latex=b_s#0)) affect calling, where [
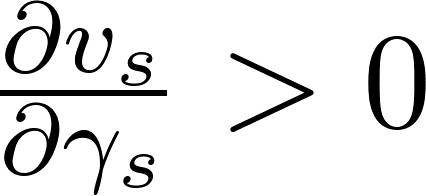
](https://www.codecogs.com/eqnedit.php?latex=%5Cfrac%7B%20%5Cpartial%20v_s%20%7D%7B%5Cpartial%20%5Cgamma_s%20%7D%20%3E%200#0) for all [
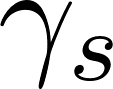
](https://www.codecogs.com/eqnedit.php?latex=%5Cgamma_s#0).

A sender with threshold [
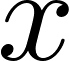
](https://www.codecogs.com/eqnedit.php?latex=x#0) has probability [
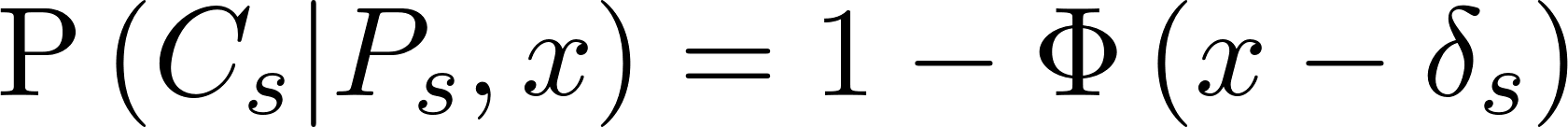
](https://www.codecogs.com/eqnedit.php?latex=%5Cmathrm%7BP%7D%5Cleft(C_s%7CP_s%2Cx%5Cright)%3D1-%5CPhi%20%20%5Cleft(x-%5Cdelta%20_s%5Cright)#0) for fleeing/calling when one of their predators is present, and [
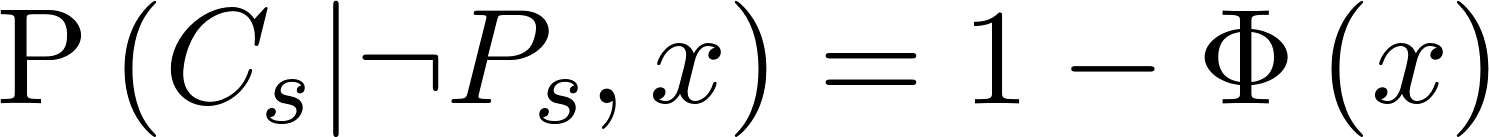
](https://latex-staging.easygenerator.com/eqneditor/editor.php?latex=%5Cmathrm%7BP%7D%5Cleft(C_s%7C%5Clnot%20P_s%2Cx%5Cright)%3D1-%5CPhi%20%20%5Cleft(x)#0) when a predator is absent. Here [
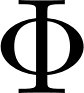
](https://www.codecogs.com/eqnedit.php?latex=%5CPhi#0) is the standard normal cumulative distribution function. With probability [
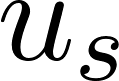
](https://www.codecogs.com/eqnedit.php?latex=u_s#0) the sender calls when a predator is detected, gaining fitness: [
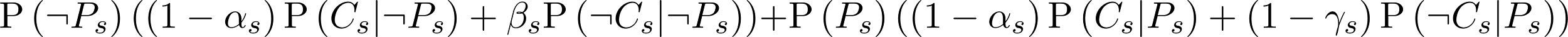
](https://www.codecogs.com/eqnedit.php?latex=%5Cmathrm%7BP%7D%5Cleft(%5Cneg%20P_s%5Cright)%20%5Cleft(%5Cleft(1-%5Calpha%20_s%5Cright)%20%5Cmathrm%7BP%7D%5Cleft(C_s%7C%5Cneg%20P_s%5Cright)%2B%5Cbeta%20_s%20%5Cmathrm%7BP%7D%5Cleft(%5Cneg%20C_s%7C%5Cneg%20P_s%5Cright)%5Cright)%2B%5Cmathrm%7BP%7D%5Cleft(P_s%5Cright)%20%5Cleft(%5Cleft(1-%5Calpha%20_s%5Cright)%20%5Cmathrm%7BP%7D%5Cleft(C_s%7CP_s%5Cright)%2B%5Cleft(1-%5Cgamma%20_s%5Cright)%20%5Cmathrm%7BP%7D%5Cleft(%5Cneg%20C_s%7CP_s%5Cright)%5Cright)#0)

Sender’s may have the strategy of not calling to detections, and with probability [
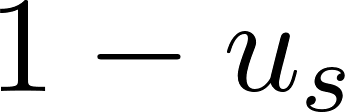
](https://www.codecogs.com/eqnedit.php?latex=1-u_s#0) they have fitness [
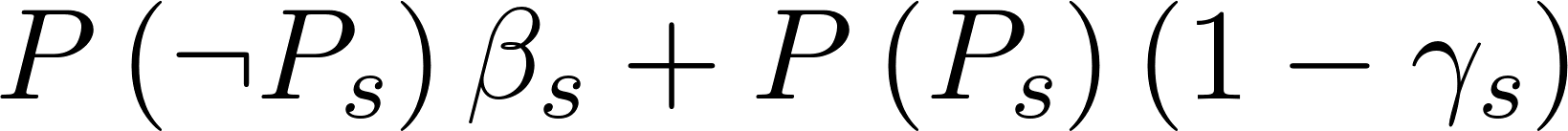
](https://latex-staging.easygenerator.com/eqneditor/editor.php?latex=P%5Cleft(%5Cneg%20P_s%5Cright)%5Cbeta%20_s%20%2BP%5Cleft(P_s%5Cright)%20%5Cleft(1-%5Cgamma%20_s%5Cright)#0). The fitness of a focal sender, [
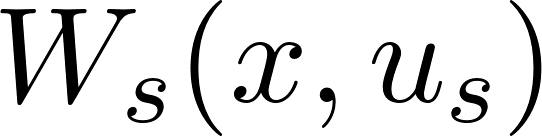
](https://www.codecogs.com/eqnedit.php?latex=W_s(x%2Cu_s)#0), is the expected value of both their calling strategy and detection threshold.

We now seek the values [
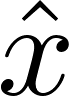
](https://www.codecogs.com/eqnedit.php?latex=%5Chat%7Bx%7D#0) and [
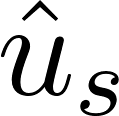
](https://www.codecogs.com/eqnedit.php?latex=%5Chat%7Bu%7D_s#0) that selection will drive sender’s trait values towards. Solving [
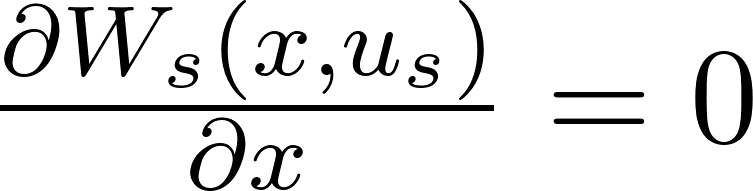
](https://www.codecogs.com/eqnedit.php?latex=%5Cfrac%7B%20%5Cpartial%20W_s(x%2Cu_s)%20%7D%7B%5Cpartial%20x%20%7D%3D%200#0) for [
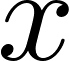
](https://www.codecogs.com/eqnedit.php?latex=x#0) yields eqn 1 in the manuscript,

|  | [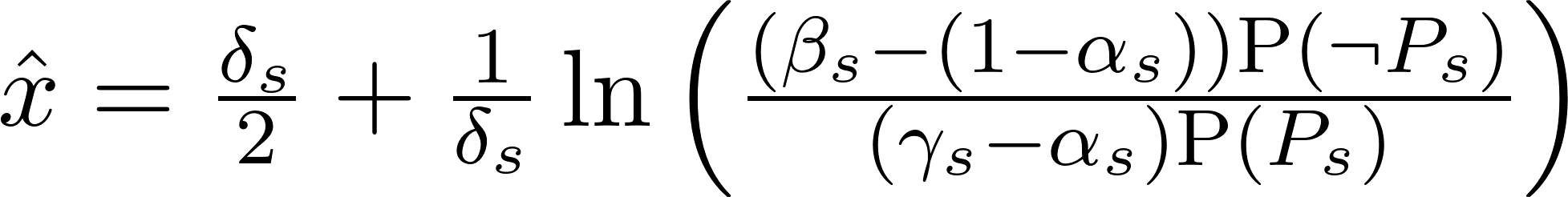](https://www.codecogs.com/eqnedit.php?latex=%20%5Chat%7Bx%7D%20%3D%20%5Cfrac%7B%5Cdelta_s%7D%7B2%7D%20%2B%20%5Cfrac%7B1%7D%7B%5Cdelta_s%7D%5Cln%7B%5Cleft(%5Cfrac%7B(%5Cbeta_s-(1-%5Calpha_s))%5Cmathrm%7BP%7D(%5Clnot%20P_s)%7D%7B(%5Cgamma_s-%5Calpha_s)%5Cmathrm%7BP%7D(P_s)%7D%5Cright)%7D#0) | (eqn 1) |
| --- | --- | --- |

Note,

| [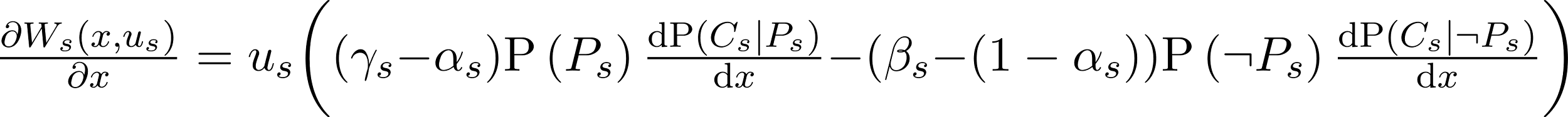](https://www.codecogs.com/eqnedit.php?latex=%5Cfrac%7B%20%5Cpartial%20W_s(x%2Cu_s)%20%7D%7B%5Cpartial%20x%20%7D%3D%20u_s%5Cbigg((%5Cgamma%20_s-%5Calpha%20_s)%5Cmathrm%7BP%7D%5Cleft(P_s%5Cright)%5Cfrac%7B%5Cmathrm%7Bd%7D%5Cmathrm%7BP%7D%5Cleft(C_s%7CP_s%5Cright)%7D%7B%5Cmathrm%7Bd%7Dx%7D%20-%20(%5Cbeta%20_s-%5Cleft(1-%5Calpha%20_s%5Cright))%5Cmathrm%7BP%7D%5Cleft(%5Clnot%20P_s%5Cright)%5Cfrac%7B%5Cmathrm%7Bd%7D%5Cmathrm%7BP%7D%5Cleft(C_s%7C%5Cneg%20P_s%5Cright)%7D%7B%5Cmathrm%7Bd%7Dx%7D%20%5Cbigg)#0) | (eqn SM1) |
| --- | --- |

It can be shown as a special case of the Leibniz integral that,

|  | [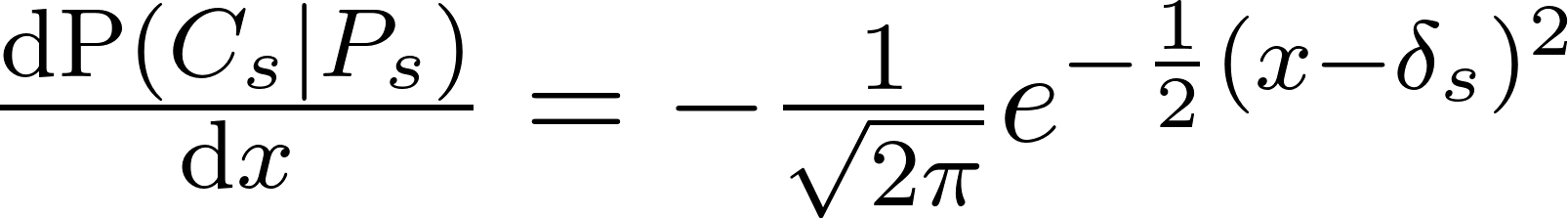](https://www.codecogs.com/eqnedit.php?latex=%5Cfrac%7B%5Cmathrm%7Bd%7D%5Cmathrm%7BP%7D%5Cleft(C_s%7CP_s%5Cright)%7D%7B%5Cmathrm%7Bd%7Dx%7D%20%3D%20%20-%20%5Cfrac%7B1%7D%7B%5Csqrt%7B2%5Cpi%7D%7De%5E%7B-%5Cfrac%7B1%7D%7B2%7D(x%20-%20%5Cdelta_s)%5E%7B2%7D%7D#0) | (eqn SM2) |
| --- | --- | --- |

Similarly, [
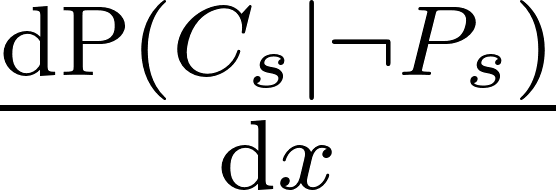
](https://www.codecogs.com/eqnedit.php?latex=%5Cfrac%7B%5Cmathrm%7Bd%7D%5Cmathrm%7BP%7D%5Cleft(C_s%7C%5Cneg%20P_s%5Cright)%7D%7B%5Cmathrm%7Bd%7Dx%7D#0) has the same functional form but with [
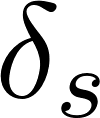
](https://www.codecogs.com/eqnedit.php?latex=%5Cdelta_s#0) = 0.

As [
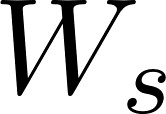
](https://www.codecogs.com/eqnedit.php?latex=W_s#0) is a linear function of [
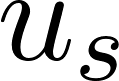
](https://www.codecogs.com/eqnedit.php?latex=u_s#0), finding when [
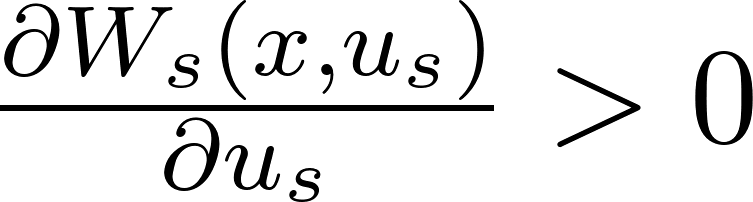
](https://www.codecogs.com/eqnedit.php?latex=%5Cfrac%7B%20%5Cpartial%20W_s(x%2Cu_s)%20%7D%7B%5Cpartial%20u_s%20%7D%20%3E%200#0) yields the condition under which calling will have convergence and evolutionarily stable strategy, [
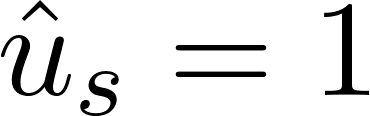
](https://www.codecogs.com/eqnedit.php?latex=%5Chat%7Bu%7D_s%20%3D%201#0). Because our model contains no frequency or density dependence, convergence and evolutionarily stability is equivalent to simple optimality by the second derivative test. This condition is:

|  | [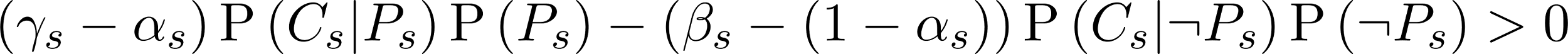](https://www.codecogs.com/eqnedit.php?latex=%5Cleft(%5Cgamma%20_s-%5Calpha%20_s%5Cright)%20%5Cmathrm%7BP%7D%5Cleft(C_s%7CP_s%5Cright)%5Cmathrm%7BP%7D%5Cleft(P_s%5Cright)-%5Cleft(%5Cbeta%20_s-%5Cleft(1-%5Calpha%20_s%5Cright)%5Cright)%20%5Cmathrm%7BP%7D%5Cleft(C_s%7C%5Cneg%20P_s%5Cright)%5Cmathrm%7BP%7D%5Cleft(%5Cneg%20P_s%5Cright)%3E0#0) | (eqn SM3) |
| --- | --- | --- |

In testing stability, it can be shown that when [
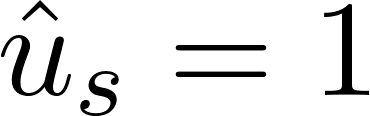
](https://www.codecogs.com/eqnedit.php?latex=%5Chat%7Bu%7D_s%20%3D%201#0) that [
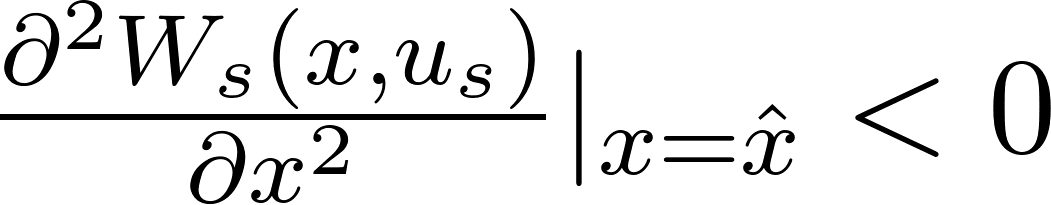
](https://latex-staging.easygenerator.com/eqneditor/editor.php?latex=%20%5Cfrac%7B%20%5Cpartial%5E2%20W_s(x%2Cu_s)%20%7D%7B%5Cpartial%20x%5E2%20%7D%5Crvert_%7Bx%20%3D%20%5Chat%7Bx%7D%7D%3C0#0). This shows [
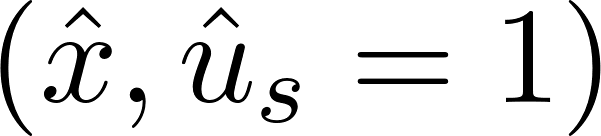
](https://www.codecogs.com/eqnedit.php?latex=(%5Chat%7Bx%7D%2C%20%5Chat%7Bu%7D_s%20%3D1%20)#0) has convergence and evolutionarily stable strategy in the current model (there is only a single equilibrium threshold value [
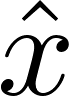
](https://www.codecogs.com/eqnedit.php?latex=%5Chat%7Bx%7D#0)). Therefore, in our analysis of eavesdropping, we assume that the sender calls on the threshold [
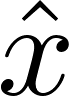
](https://www.codecogs.com/eqnedit.php?latex=%5Chat%7Bx%7D#0).

Note, [
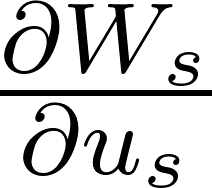
](https://www.codecogs.com/eqnedit.php?latex=%5Cfrac%7B%20%5Cpartial%20W_s%20%7D%7B%5Cpartial%20u_s%20%7D#0) and [
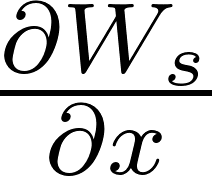
](https://www.codecogs.com/eqnedit.php?latex=%5Cfrac%7B%20%5Cpartial%20W_s%20%7D%7B%5Cpartial%20x%20%7D#0) are fitness gradients, telling us for any specific [
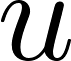
](https://www.codecogs.com/eqnedit.php?latex=u#0) and [
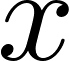
](https://www.codecogs.com/eqnedit.php?latex=x#0) if selection favours this strategy/trait value, under the condition the fitness gradient is positive. In the general case, selection takes the populations strategy towards the optimum value that exists where fitness is maximised (e.g., [
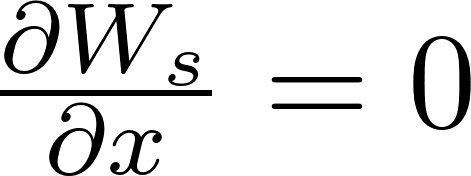
](https://www.codecogs.com/eqnedit.php?latex=%5Cfrac%7B%20%5Cpartial%20W_s%20%7D%7B%5Cpartial%20x%20%7D%3D0#0), [
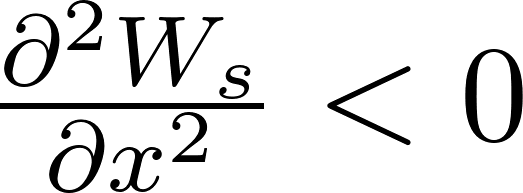
](https://www.codecogs.com/eqnedit.php?latex=%5Cfrac%7B%20%5Cpartial%5E2%20W_s%20%7D%7B%5Cpartial%20x%5E2%20%7D%3C0#0) ). In the case of a monotonic strategy such as [
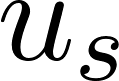
](https://www.codecogs.com/eqnedit.php?latex=u_s#0), the optimal behaviour technically exists at the boundary (namely, [
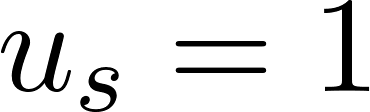
](https://www.codecogs.com/eqnedit.php?latex=u_s%20%3D%201#0) or [
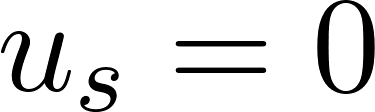
](https://www.codecogs.com/eqnedit.php?latex=u_s%20%3D%200#0)).

1. **FURTHER INFORMATION: CONDITIONS FOR SELECTION OF EAVESDROPPING**

A receiver species may have different fitness payoffs for fleeing [
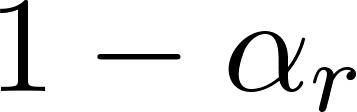
](https://www.codecogs.com/eqnedit.php?latex=1-%5Calpha_r#0), remaining and possibility encountering one of their own predators [
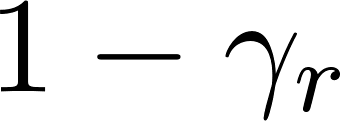
](https://latex-staging.easygenerator.com/eqneditor/editor.php?latex=1-%5Cgamma_r#0), as well as benefiting from remaining in the absence of a predator [
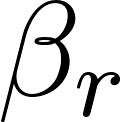
](https://latex-staging.easygenerator.com/eqneditor/editor.php?latex=%5Cbeta_r#0). A potential eavesdropping receiver must rely on the sender's calls for predator detection. Receivers can either heed calls and flee, with probability [
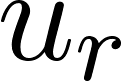
](https://latex-staging.easygenerator.com/eqneditor/editor.php?latex=u_r#0), or remain when a call is detected, [
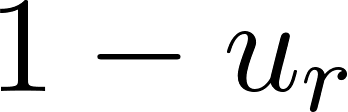
](https://www.codecogs.com/eqnedit.php?latex=1-u_r#0). Receivers vary in the threshold [
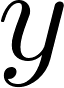
](https://latex-staging.easygenerator.com/eqneditor/editor.php?latex=y#0) they set for the amount of evidence of a call they require to flee based on the sender’s calls. Again, we assume an equal variance Gaussian model, where the receiver’s sensitivity index is [
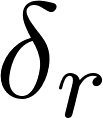
](https://www.codecogs.com/eqnedit.php?latex=%5Cdelta_r#0). Let [
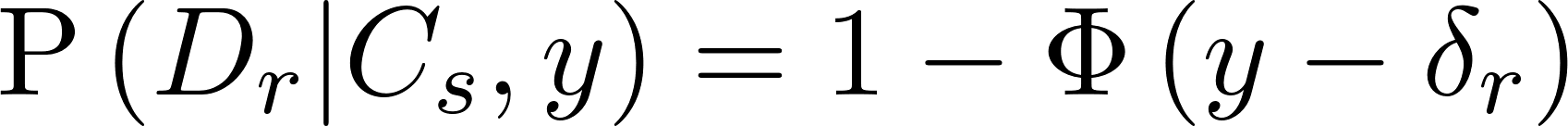
](https://www.codecogs.com/eqnedit.php?latex=%5Cmathrm%7BP%7D%5Cleft(D_r%7CC_s%2Cy%5Cright)%3D1-%5CPhi%20%20%5Cleft(y-%5Cdelta%20_r%5Cright)#0) be the probability of detecting a call when one is present, and [
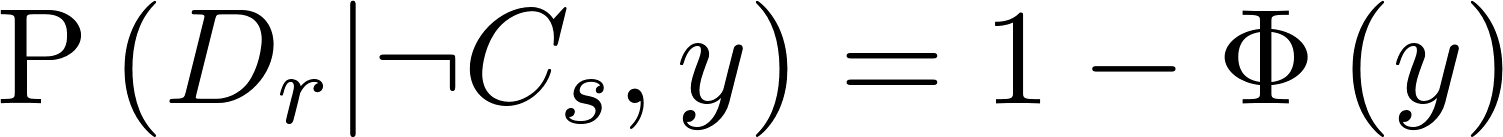
](https://latex-staging.easygenerator.com/eqneditor/editor.php?latex=%5Cmathrm%7BP%7D%5Cleft(D_r%7C%5Clnot%20C_s%2Cy%5Cright)%3D1-%5CPhi%20%20%5Cleft(y)#0) of detecting a call when no call is present.

The different combinations of events that impact the receiver’s fitness are: (1) a predator of the sender is present or absent ([
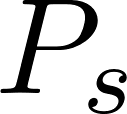
](https://www.codecogs.com/eqnedit.php?latex=P_s#0) or [
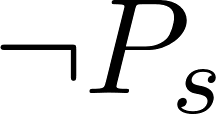
](https://www.codecogs.com/eqnedit.php?latex=%5Cneg%20P_s#0)), (2) a predator of the receiver is present or absent ([
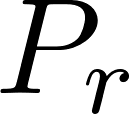
](https://www.codecogs.com/eqnedit.php?latex=P_r#0) or [
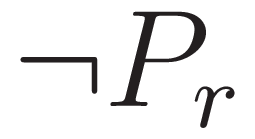
](http://www.sciweavers.org/tex2img.php?bc=Transparent&fc=Black&im=jpg&fs=100&ff=modern&edit=0&eq=%5Cneg%20P_r#0)), (3) the sender called or was silent ([
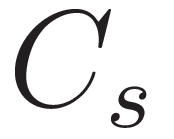
](http://www.sciweavers.org/tex2img.php?bc=Transparent&fc=Black&im=jpg&fs=100&ff=modern&edit=0&eq=C_s#0) or [
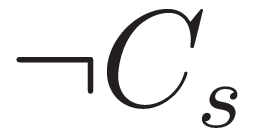
](http://www.sciweavers.org/tex2img.php?bc=Transparent&fc=Black&im=jpg&fs=100&ff=modern&edit=0&eq=%5Cneg%20C_s#0)), (4) receiver correctly detected or missed the call ([
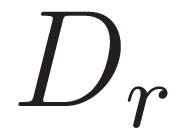
](http://www.sciweavers.org/tex2img.php?bc=Transparent&fc=Black&im=jpg&fs=100&ff=modern&edit=0&eq=D_r#0) or [
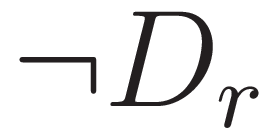
](http://www.sciweavers.org/tex2img.php?bc=Transparent&fc=Black&im=jpg&fs=100&ff=modern&edit=0&eq=%5Cneg%20D_r#0)). Furthermore, if the receiver detects a call they may flee to calls (eavesdropping) or not, with probability [
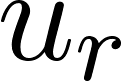
](https://latex-staging.easygenerator.com/eqneditor/editor.php?latex=u_r#0) and [
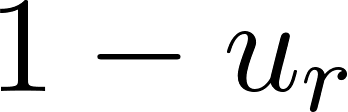
](https://www.codecogs.com/eqnedit.php?latex=1-%20u_r#0), respectively. However, if they do not detect a call they will definitely remain. For completeness, Table S2 provides an exhaustive list of the probability of different combinations of events occurring and the associated payoff for the receiver. We postpone discussion of personal detection until later. The fitness of a focal receiver, [
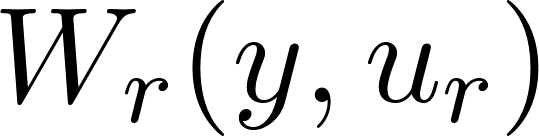
](https://www.codecogs.com/eqnedit.php?latex=W_r(y%2Cu_r)#0) is the expected value of these subsets of events.

**TABLE S2** Subsets of events affecting the fitness of the receiver, and the associated payoffs

| Probability of Subset of Events | | | | | Payoff |
| --- | --- | --- | --- | --- | --- |
| [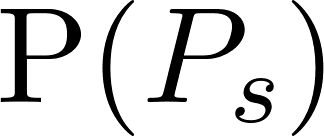](https://www.codecogs.com/eqnedit.php?latex=%5Cmathrm%7BP%7D(P_s)#0) | [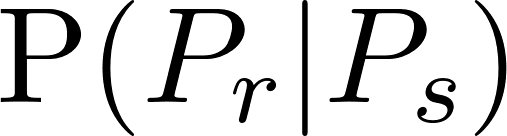](https://www.codecogs.com/eqnedit.php?latex=%5Cmathrm%7BP%7D(P_r%20%7C%20P_s)#0) | [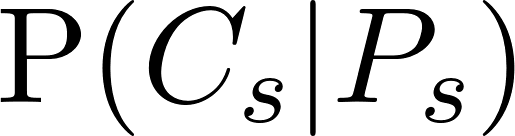](https://www.codecogs.com/eqnedit.php?latex=%5Cmathrm%7BP%7D(C_s%20%7C%20P_s)#0) | [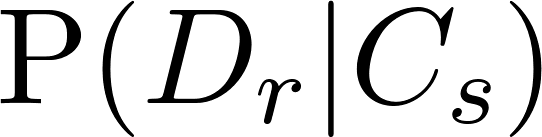](https://latex-staging.easygenerator.com/eqneditor/editor.php?latex=%5Cmathrm%7BP%7D(D_r%20%7C%20C_s)#0) | [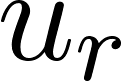](https://latex-staging.easygenerator.com/eqneditor/editor.php?latex=u_r#0) | [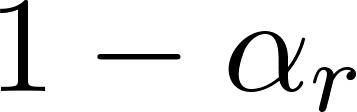](https://www.codecogs.com/eqnedit.php?latex=1-%5Calpha_r#0) |
| [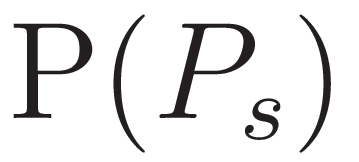](http://www.sciweavers.org/tex2img.php?bc=Transparent&fc=Black&im=jpg&fs=100&ff=modern&edit=0&eq=%5Cmathrm%7BP%7D(P_s)#0) | [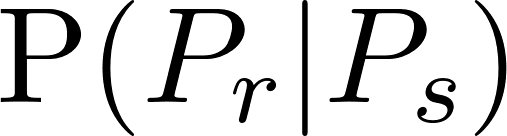](https://www.codecogs.com/eqnedit.php?latex=%5Cmathrm%7BP%7D(P_r%20%7C%20P_s)#0) | [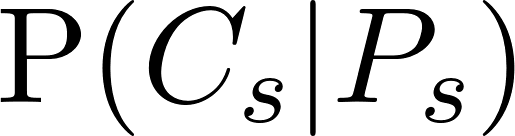](https://www.codecogs.com/eqnedit.php?latex=%5Cmathrm%7BP%7D(C_s%20%7C%20P_s)#0) | [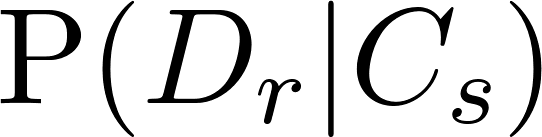](https://latex-staging.easygenerator.com/eqneditor/editor.php?latex=%5Cmathrm%7BP%7D(D_r%20%7C%20C_s)#0) | [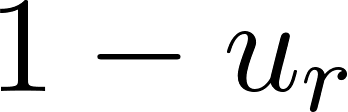](https://www.codecogs.com/eqnedit.php?latex=1-%20u_r#0) | [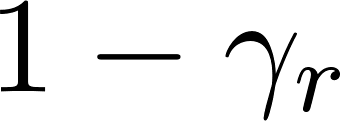](https://latex-staging.easygenerator.com/eqneditor/editor.php?latex=1-%5Cgamma_r#0) |
| [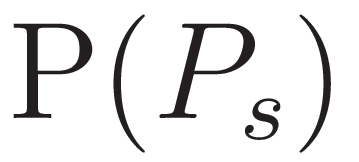](http://www.sciweavers.org/tex2img.php?bc=Transparent&fc=Black&im=jpg&fs=100&ff=modern&edit=0&eq=%5Cmathrm%7BP%7D(P_s)#0) | [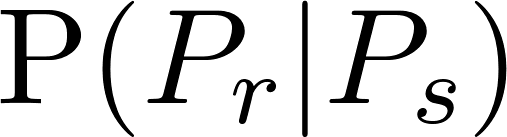](https://latex-staging.easygenerator.com/eqneditor/editor.php?latex=%5Cmathrm%7BP%7D(P_r%20%7C%20P_s)#0) | [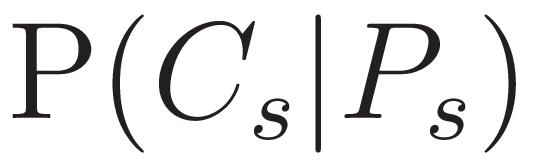](http://www.sciweavers.org/tex2img.php?bc=Transparent&fc=Black&im=jpg&fs=100&ff=modern&edit=0&eq=%5Cmathrm%7BP%7D(C_s%20%7C%20P_s)#0) | [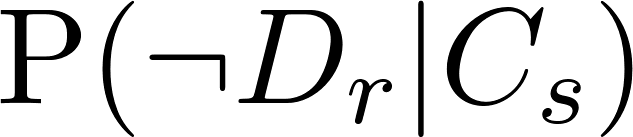](https://latex-staging.easygenerator.com/eqneditor/editor.php?latex=%5Cmathrm%7BP%7D(%5Cneg%20D_r%20%7C%20C_s)#0) |  | [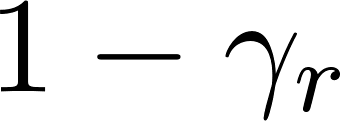](https://latex-staging.easygenerator.com/eqneditor/editor.php?latex=1-%5Cgamma_r#0) |
| [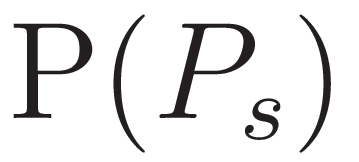](http://www.sciweavers.org/tex2img.php?bc=Transparent&fc=Black&im=jpg&fs=100&ff=modern&edit=0&eq=%5Cmathrm%7BP%7D(P_s)#0) | [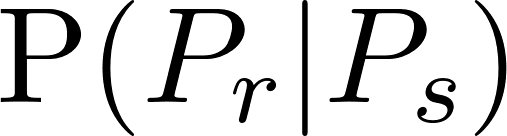](https://www.codecogs.com/eqnedit.php?latex=%5Cmathrm%7BP%7D(P_r%20%7C%20P_s)#0) | [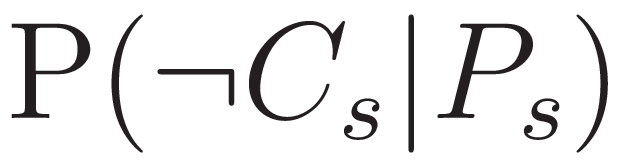](http://www.sciweavers.org/tex2img.php?bc=Transparent&fc=Black&im=jpg&fs=100&ff=modern&edit=0&eq=%5Cmathrm%7BP%7D(%5Cneg%20C_s%20%7C%20P_s)#0) | [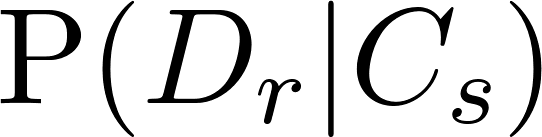](https://latex-staging.easygenerator.com/eqneditor/editor.php?latex=%5Cmathrm%7BP%7D(D_r%20%7C%20C_s)#0) | [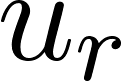](https://latex-staging.easygenerator.com/eqneditor/editor.php?latex=u_r#0) | [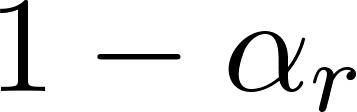](https://www.codecogs.com/eqnedit.php?latex=1-%5Calpha_r#0) |
| [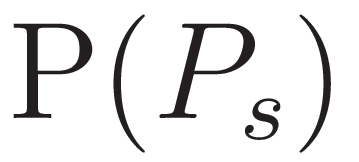](http://www.sciweavers.org/tex2img.php?bc=Transparent&fc=Black&im=jpg&fs=100&ff=modern&edit=0&eq=%5Cmathrm%7BP%7D(P_s)#0) | [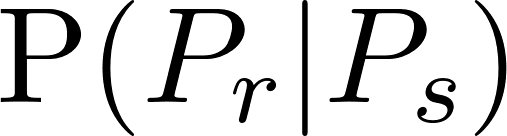](https://www.codecogs.com/eqnedit.php?latex=%5Cmathrm%7BP%7D(P_r%20%7C%20P_s)#0) | [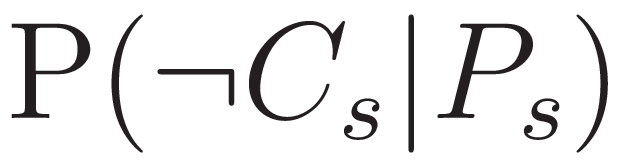](http://www.sciweavers.org/tex2img.php?bc=Transparent&fc=Black&im=jpg&fs=100&ff=modern&edit=0&eq=%5Cmathrm%7BP%7D(%5Cneg%20C_s%20%7C%20P_s)#0) | [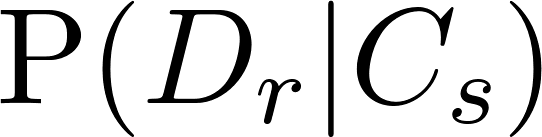](https://latex-staging.easygenerator.com/eqneditor/editor.php?latex=%5Cmathrm%7BP%7D(D_r%20%7C%20C_s)#0) |  |  |
|  |  |  |  |  |  |
|  |  |  |  |  |  |
|  |  |  |  |  |  |
|  |  |  |  |  |  |
|  |  |  |  |  |  |
|  |  |  |  |  |  |
|  |  |  |  |  |  |
|  |  |  |  |  |  |
|  |  |  |  |  |  |
|  |  |  |  |  |  |
|  |  |  |  |  |  |
|  |  |  |  |  |  |
|  |  |  |  |  |  |
|  |  |  |  |  |  |
|  |  |  |  |  |  |
|  |  |  |  |  |  |
|  |  |  |  |  |  |
|  |  |  |  |  |  |
|  |  |  |  |  |  |

As with selection for calling in senders, eavesdropping will be selected when , this is the same as enq 2 in the main text:

|  | (eqn 2) |
| --- | --- |
|  |  |
|  |  |
|  |  |
|  |  |

Note, the net benefit of avoiding predation for the receiver is , and the net cost of unnecessary fleeing is .

Enq 2 is the key condition of interest in the current paper, when it is fulfilled eavesdropping is associated with higher fitness than not eavesdropping, and selection will take the population towards fixation for eavesdropping; that is,  will have convergence and evolutionarily stable. We assume a population of receivers who have not had extensive selection for eavesdropping, so there are a small number of eavesdroppers and their call detection threshold has not been selected. Consequently, the call detection threshold has not approached a selected maximum , so we treat  as a parameter to see how it influences selection for eavesdropping. This assumption is made for simplicity.

We can examine how our parameters affect selection for eavesdropping by seeing the effect they have on its fitness gradient  (as reflected in Inq 2):

|  |  | (eqn SM4) |
| --- | --- | --- |

When an increase in a parameter is associated with an increase in the fitness gradient, the parameter makes the evolution of eavesdropping more likely. This is because an increase in the parameter is associated with an increase in the relative fitness of eavesdroppers compared to non-eavesdroppers. Therefore, we can check when a parameter selects for eavesdropping by examining when the fitness gradient with respect to that parameter is positive.

1. **FURTHER INFORMATION: PREDATOR RELEVANCE**

Selection for eavesdropping has a single maxima as a function of the sender’s threshold, which exists when the relative vulnerability of sender and receiver are identical. This selection for eavesdropping maximising threshold, , can be found by setting the following derivative of the eavesdropping fitness gradient with respect to the sender’s threshold equal to 0 and solving:

|  | (eqn SM5) |
| --- | --- |

1. **FURTHER INFORMATION: SENDER DISCRIMINATION**

The fitness gradient of eavesdropping with respect to discrimination is complicated and yields little direct insight. Therefore, we predominately gained an understanding of the effect of discrimination by parameter-sweep investigations. However, note the general form of the fitness gradient is:

|  | (eqn SM6) |
| --- | --- |

When discrimination is better the sender will produce more correct detections of shared predators, leading to more predator evasion by the receiver. Therefore, the primary effect of superior discrimination is to increase selection for eavesdropping, . This primary effect more strongly selects eavesdropping when the receiver is highly vulnerable to predators of the sender and these predators are likely to be encountered. Further, there is a stronger primary effect when reception is good. There is also a secondary effect changes in discrimination have on eavesdropping via discrimination’s direct effect on the sender’s threshold for calling, .

While under the majority of parameter combinations superior discrimination leads to greater selection for eavesdropping, it is theoretically possible for improving discrimination to select against eavesdropping. This occurs because of the secondary effect discrimination has on eavesdropping, via selection on the sender’s calling threshold. Specifically, when discrimination is poor, the cost of errors have an amplified effect on the adaptive biasing of the sender’s calling threshold; small differences in costs of false alarms versus missed detections select for more extreme fastidiousness or gullibility. When sender and receiver differ substantially in vulnerability to shared predators, it could hypothetically occur that extreme adaptive biasing ensures the sender calls on a threshold that is favourable for the receiver; even though differences in sender and receiver vulnerability mean that if discrimination was better eavesdropping would not be favoured. In such cases, small improvements from very poor to moderately poor discrimination can select against eavesdropping. However, large improvements will still select for eavesdropping because of the overwhelming effect of increasing correctly detecting shared predators. We expect this situation to be unusual.

1. **FURTHER INFORMATION: RECEIVER ALARM CALL DETECTION THRESHOLD**

Selection for eavesdropping is strongest at the intermediate values of receiver threshold, being maximised at the point:

|  |  | (eqn SM7) |
| --- | --- | --- |

1. **FURTHER INFORMATION: PERSONAL INFORMATION**

We extend our model to the situation in which a receiver may gain personal cues to the presence of a predator along with cues of a call from a sender species. The receiver flees if either of these cues indicates a predator, and remains if neither cue indicates a predator. The full fitness function is cumbersome and adds more terms to Table S2. For instance, the expected fitness of fleeing, given the receiver eavesdrops when one of their own predators is present is:

|  |  | (eqn SM8) |
| --- | --- | --- |

Whereas, the expected fitness of fleeing given the receiver does not eavesdrop is:

|  |  | (eqn SM9) |
| --- | --- | --- |

We provide the key condition for eavesdropping, allowing the receiver to access personal information, in the main text.

1. **FURTHER INFORMATION: DEPLETED RESERVES CAUSED BY REPEATED FLEEING**

We extended the assumptions of McNamara and Trimmer (2019) to the case of an eavesdropping receiver. In particular, the probability of regaining a unit of energy reserve levels for the receiver is:

|  |  | (eqn SM10) |
| --- | --- | --- |

This is analogous in form to the probability of regaining a unit of energy for the sender (McNamara & Trimmer, 2019); however, the receiver’s probability of remaining, , and the probability of fleeing, , are dependent on the behaviour of the sender (e.g., whether the sender calls), as well as the presence of unique predators. The sender () and receiver () may differ in mortality from sources other than the predator, and the payoff gained by foraging may also differ between species ( may differ from ). However, to simplify and focus on whether repeated encounters with predators effect eavesdropping, we assumed prey species were similar in foraging success and morality due to sources other than predation, as well as assuming reception and discrimination were effective (Figure 4, main text).
